# Food Properties, Bioactive Components, and Human Factors as Determinants of Low Nutrient Supply From Foods Can Be Improved With Adequate Nutritional Strategies

**DOI:** 10.1002/fsn3.72528

**Published:** 2026-11-01

**Authors:** Chinaza Godswill Awuchi

**Affiliations:** ^1^ School of Natural and Applied Sciences, Kampala International University Kampala Uganda; ^2^ UNESCO Encyclopedia of Life Support Systems UNESCO EOLSS Publishers Paris France

**Keywords:** antinutritional factors, bioaccessibility, bioavailability, food processing, food security, fortification, nutrient adequacy, nutrition policy

## Abstract

Nutrient adequacy depends not only on the nutrient content of foods but also on food‐matrix release, intestinal absorption, physiological utilization, dietary access, and the stability of food supplies. These stages are influenced by antinutritional compounds, processing, storage, microbial activity, agricultural conditions, disease, genetics, and socioeconomic constraints. This narrative review critically synthesizes food‐level, host‐level, and food system determinants of nutrient supply and evaluates strategies for addressing them. Relevant literature was sought from PubMed/MEDLINE, PubMed Central (PMC), Scopus, Web of Science, Embase, the Cochrane Library, ScienceDirect, etc. A narrative/critical synthesis was organized around nutrient content, bioaccessibility, bioavailability, utilization, and population access. Evidence was interpreted according to study design and applicability, distinguishing mechanistic and in vitro findings from human absorption studies, clinical outcomes, and program‐level evidence. Processing and bioactive compounds can either inhibit or improve nutrient availability depending on dose, food matrix, and preparation. Host conditions and structural barriers may prevent nutrient‐rich foods from translating into adequate nutrient status. Fortification and supplementation can be effective for defined deficiencies, but coverage, safety, affordability, dietary diversity, and monitoring determine impact. Emerging technologies require stronger evidence on human outcomes, safety, and cost‐effectiveness before broad implementation. Effective action requires context‐specific combinations of food processing, safe storage, dietary diversification, agricultural improvement, targeted nutrition services, and equitable food system policies.

## Graphical abstract

Nutrient adequacy depends on food composition, bioaccessibility, absorption, physiological utilization, dietary access, supply stability, etc. Food properties, human health, agricultural conditions, and socioeconomic barriers influence nutrient delivery. Improved processing, safe storage, dietary diversification, agricultural improvement, targeted nutrition services, and equitable policies strengthen nutrient status, health outcomes, and food security.

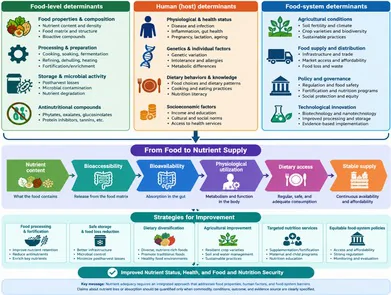

## 1 Introduction

Low nutrient supply from foods has been a major challenge facing food and nutrition security in many regions, especially in the developing and underdeveloped world. Undernutrition is most likely when individuals get insufficient nutrients from foods, and often results in many health problems, including weak immune system, cardiovascular diseases, nutrient deficiency diseases, infections, muscle weakness, nervous system disorders, etc. Low nutrient supply often leads to nutrient deficiencies and imbalance of protein and energy, which have adverse effects on body tissues and structure. Prolonged low supply of nutrients from foods can lead to stunted growth, wasting, hidden hunger, and underweight (Swinburn et al. 2019). Millions of people worldwide suffer from one or more forms of undernutrition, such as stunting, wasting, mineral deficiency, vitamin deficiency, or noncommunicable diseases associated with low dietary nutrients (Murarkar et al. 2020). Undernutrition is more common in developing countries. Studies on low nutrient supply from diets usually categorize the population into various groups such as infants, children below 5, adolescents, children, lactating mothers, pregnant women, adults, and elderly, and concluded that the prevalence of undernutrition resulting from low nutrient supply is highest among children below five (Murarkar et al. 2020). The deficiency of vitamin A affects one out of three children below age 5 around the world. In general, developing and underdeveloped nations are more vulnerable to poor nutrition and low nutrient supply from foods (Our World in Data 2021).

Nutritional inadequacy is not explained by food availability or nutrient composition alone (Awuchi 2026a, 2026b, 2026c). A food may contain substantial quantities of a nutrient while providing little of it in a form that is released from the matrix, absorbed, transported, or used physiologically. Conversely, processing can reduce the concentration of heat‐sensitive nutrients while increasing digestibility, reducing selected antinutritional factors, or improving microbiological safety. Accordingly, “low nutrient supply” should be treated as an outcome of interacting food, host, and food‐system processes rather than as a synonym for low nutrient content. Undernutrition remains a major public‐health concern, but prevalence estimates are time‐ and population‐specific and should be cited to the relevant surveillance year rather than treated as current indefinitely (Murarkar et al. 2020; Swinburn et al. 2019).

Food insecurity and poor dietary quality are shaped by poverty, conflict, climate variability, food prices, unequal distribution, and gaps in health services. These determinants affect whether people can obtain diverse foods, store and prepare them safely, and access care for conditions that impair digestion or metabolism. Global and regional statistics therefore describe different points in a causal pathway and should not be interpreted as proof that low nutrient supply alone caused a given health outcome. Food‐system losses are also heterogeneous by commodity and region; the Food and Agriculture Organization's Food Loss Index reports variation across commodity groups and regions rather than supporting a universal percentage for all foods (Food and Agriculture Organization of the United Nations 2025). The WHO (2021) reported that 149.2 million children below 5 were stunted, 45.4 million wasted, while 462 million are underweight. All these are as a result of poor nutrient supply from foods (WHO 2021). There is increase in hunger worldwide in recent decade. Around 795 million people (one tenth of the world population) had undernutrition in 2015. This was recently made worse by the COVID‐19 pandemic and its associated lockdown worldwide (Nyarko et al. 2022; Egbuna, Archibong, et al. 2022; Egbuna, Chandra, et al. 2022). The Global Nutrition Report released in early 2022 reported that, in 2021, 820 million people was hungry worldwide, i.e., 1/9 people worldwide (Global Nutrition Report 2022). The report also stated that, of the 194 countries, over a quarter (49 countries) are on course to meet nutrition targets leading to stunting and wasting targets. Anemia prevalence due to nutrition insecurity are showing no progress or worsening in more 161 countries. In the Africa region, no country is on course to meet any of the diet‐related NCD targets, and the only countries on course to meet both raised blood pressure and diabetes targets are a few high‐income Western countries (Global Nutrition Report 2022). There is need for all food stakeholders to take stronger, decisive, and well‐coordinated actions to counter low nutrient supply, malnutrition, and poor dietary pattern in all parts of the world and in all forms. There should be a step forward towards understanding, planning, and acting to end poor dietary patterns and low nutrient supply from foods.

Existing reviews have examined particular components of this problem, including antinutritional factors, micronutrient bioavailability, food processing, postharvest loss, fortification, and nutrition policy. The contribution of this review is to connect these strands in a single analytical pathway—from nutrient content and release from the food matrix through absorption and utilization to affordability, access, and sustained availability. This framing also makes explicit a key limitation in the evidence base: biochemical plausibility or an in vitro change does not, by itself, establish improved absorption in humans, improved nutritional status, or a population‐level health benefit.

In rural areas, and some urban cities, the deficiencies of micronutrients, especially vitamin and mineral deficiencies (hidden hunger) are among the major causes of health issues, including noncommunicable diseases. Micronutrients' presence in bulk is not just the only nutritional factor; their bioavailability is important for human nutrient needs (Melse‐Boonstra 2020; de Juras et al. 2022). Low micronutrients' bioavailability in raw foods reduces the nutritional values of the foods and may result in the occurrence of metabolic disorders in affected population; consequently, the nutritional status of a given population can be upgraded by improving food nutrients' bioavailability (Fan et al. 2019). Most foods such as legumes comprise several anti‐nutrients or natural toxicants, which include isoflavonoids, phytic acid, cyanogens, saponins, protease inhibitors, tannins, metal chelators, etc. (Samtiya et al. 2020). Phytate is among the main anti‐nutrients and can chelate and affect bioavailability of micronutrients such as calcium, iron, copper, zinc, etc. Oxalates and polyphenols have also been described as anti‐nutrients that can limit nutrient bioavailability (Akter et al. 2020, 2022). Many phenolic compounds and other phytochemicals exert antioxidant properties through reducing power, scavenging ROS, and/or metal‐chelating actions on ferrous and ferric ions. Many anti‐nutritional factors occur in grains, such as wheat, and can affect the digestibility of nutrients derived from grain products, consequently affecting human health (FAO 2018). Anti‐nutrients have adverse effects on a food's nutritional value; as a result, detoxifying them from foods is one of the major goals in nutrition (Akter et al. 2020, 2022). In addition, many antinutrient‐containing foods such as legumes are commonly consumed as sources of protein together with cereals. Food processing employing suitable detoxification approaches for these foods should be considered before consumption to eliminate these anti‐nutrients and increase the supply of nutrients.

The objectives are to: (1) distinguish nutrient content, density, bioaccessibility, bioavailability, absorption, utilization, and dietary adequacy; (2) critically evaluate food, host, agricultural, storage, and socioeconomic determinants; (3) compare established interventions with emerging technologies in terms of evidence, feasibility, equity, safety, and cost; and (4) identify research and implementation priorities for context‐specific nutrition action. Table 1 shows selected vitamins and minerals of public health relevance: dietary sources and recommended intakes.

**TABLE 1:** Selected vitamins and minerals of public health relevance: Dietary sources and recommended intakes.

Micronutrients (vitamins and minerals)	Foods commonly found	Recommended dietary allowances for those 19–70 (female/male)	References
Vitamin A	Plant origin (as provitamin A and all‐*trans*‐beta‐carotene): milk and dairy products, fish, liver: carrots, leafy vegetables, orange, pumpkin, spinach, ripe yellow fruits, squash. Animal origin (as all‐*trans*‐Retinol and Vitamin A)	700 μg/900 μg	National Institute of Health (2013), Dalmoro et al. (2019), Doaa and Alejandro (2021)
Vitamin B_1_	Pork, brown rice, liver, potatoes, eggs, whole meal grains, vegetables	1.1 mg/1.2 mg	National Institute of Health (2013), Doaa and Alejandro (2021)
Vitamin B_2_	Milk, dairy products, bananas, asparagus, green beans	1.1 mg/1.3 mg	Dalmoro et al. (2019), Doaa and Alejandro (2021)
Vitamin B_3_	Meat, fish, eggs, tree nuts, mushrooms	14 mg/16 mg	Dalmoro et al. (2019), Doaa and Alejandro (2021)
Vitamin B_5_	Meat, avocados, broccoli, etc.	5 mg for both male and female	National Institute of Health (2013), Dalmoro et al. (2019), Doaa and Alejandro (2021)
Vitamin B_6_	Meat, vegetables, bananas, tree nut	1.2 to 1.5 mg/1.3 to 1.7 mg	National Institute of Health (2013), Dalmoro et al. (2019), Doaa and Alejandro (2021)
Vitamin B_7_	liver, peanuts, raw egg yolk, leafy green vegetables	AI: 30 μg/30 μg	National Institute of Health (2013), Dalmoro et al. (2019), Doaa and Alejandro (2021)
Vitamin B_9_	Cereal, liver, leafy vegetables, bread, pasta	400 μg, male/female	National Institute of Health (2013), Dalmoro et al. (2019)
Vitamin B_12_	Meat, fish, poultry, eggs, milk	2.4 μg, male/female	National Institute of Health (2013), Doaa and Alejandro (2021)
Vitamin C	Fruits, vegetables, liver	75 mg/90 mg	National Institute of Health (2013), Dalmoro et al. (2019)
Vitamin D	Liver, some mushroom (shiitake), some fish species (sardines), eggs	15 μg, male/female	National Institute of Health (2013)
Vitamin E	Fruits, seed oils, seeds, nuts, vegetables	15 mg, male/female	(Dalmoro et al. 2019, Doaa and Alejandro 2021)
Vitamin K	Leafy green vegetables, egg yolks, liver	AI: 120 μg/110 μg	National Institute of Health (2013), Dalmoro et al. (2019), Doaa and Alejandro (2021)
Arsenic	Drinking water, mushroom, fish	NE	National Institute of Health (2013), Dalmoro et al. (2019), Doaa and Alejandro (2021)
Boron	Apples, milk, coffee, beans, potatoes, fruits, fruit juices, cheese, leafy green vegetables, grains	NE, although some sources recommend 1–17 mg	National Institute of Health (2013), Dalmoro et al. (2019), Doaa and Alejandro (2021)
Bromine	Grains, brominated vegetable oil, grain flour, nuts, bread, seafood, sea salt	2–8 mg	National Institute of Health (2013), Dalmoro et al. (2019), Doaa and Alejandro (2021)
Calcium	Milk, canned fish with bones (salmon, sardines), dairy products, oregano, nuts, eggs, thyme, seeds, cinnamon, tofu, green leafy vegetables, dill	1000 mg	National Institute of Health (2013), Dalmoro et al. (2019), Doaa and Alejandro (2021)
Chlorine	Table salt (Sodium chloride)	2300 mg	National Institute of Health (2013), Doaa and Alejandro (2021)
Chromium	Whole grain products, broccoli, meat, grape juice	0.25–0.035 mg	National Institute of Health (2013), Dalmoro et al. (2019), Doaa and Alejandro (2021)
Cobalt	Considered part of vitamin B_12_ (cobalamins) obtained from animal sources	NE	Doaa and Alejandro (2021)
Copper	Seafood, oysters, liver, nuts, seeds, whole grains	0.9 mg	Dalmoro et al. (2019), Doaa and Alejandro (2021)
Fluorine	Drinking water, raisins, spinach, coffee, shrimp, potatoes, black tea, grapes	0.1–3 mg	National Institute of Health (2013), Dalmoro et al. (2019)
Iodine	Iodized salt, seaweed (kelp, kombu), grains, eggs	0.150 mg	Dalmoro et al. (2019), Doaa and Alejandro (2021)
Iron	Meat, dark chocolate, seafood, nuts, beans	8–18 mg	National Institute of Health (2013), Dalmoro et al. (2019), Doaa and Alejandro (2021)
Lithium	Cereals, grains, mustard, cabbage, dairy products, vegetables, kelp, fish, meat, potatoes, tomatoes, some mineral water, spices	70–600 mg	National Institute of Health (2013), Dalmoro et al. (2019), Doaa and Alejandro (2021)

*Note:* Figures are for male and female of all ages.

Abbreviation: NE, not established/available during this study.

### 1.1 Review Approach and Evidence Interpretation

This article is a narrative/critical review. The synthesis is organized thematically around food properties, processing and storage, host factors, food system constraints, and interventions. Evidence is interpreted hierarchically: controlled human studies and systematic reviews are given greater weight for claims about absorption or health outcomes; observational and program evaluations inform real‐world effectiveness; and in vitro, animal, or mechanistic studies are treated as explanatory rather than conclusive evidence of human benefit. Numerical estimates are retained only where their population, commodity, outcome, and conditions are specified; otherwise, it avoids implying universal effects.

### 1.2 Conceptual Framework: From Food Content to Nutritional Outcome

Nutrient content is the amount present in a food; nutrient density expresses that amount relative to energy or food weight. Bioaccessibility describes release from the food matrix during digestion. Bioavailability concerns absorption and subsequent physiological availability, while utilization includes metabolism and incorporation into functional processes. Dietary adequacy is a population‐ or individual‐level outcome determined by intake, requirements, losses, disease, and physiological status. Food access and stability operate upstream by determining whether a suitable food is available and consumed at all. These stages are related but not interchangeable: an intervention may increase content without improving absorption, or improve bioaccessibility without changing clinical status. This distinction guides the interpretation of evidence throughout the review.

### 1.3 Review Methods

#### 1.3.1 Review Design and Scope

This article was designed as a narrative critical review to examine how food properties, bioactive components, processing conditions, and human factors influence the supply and utilization of essential nutrients, and to evaluate nutritional strategies that may address these constraints. A narrative approach was selected because the topic spans food chemistry, food processing, gastrointestinal physiology, dietary behavior, and public health interventions, with substantial variation in study designs and outcome measures. The review emphasizes critical interpretation of mechanistic evidence alongside human intervention findings, rather than assuming that all available studies can be combined into a single quantitative estimate. The review's transparency and critical appraisal were informed by the Scale for the Assessment of Narrative Review Articles (SANRA) (Baethge et al. 2019).

#### 1.3.2 Literature Search Strategy

Relevant literature was sought from PubMed/MEDLINE, PubMed Central (PMC), Scopus, Web of Science, Embase, the Cochrane Library, ScienceDirect, Springer Nature, Wiley Online Library, Taylor & Francis Online, MDPI, and Frontiers journals. Publications and technical guidance from the World Health Organization (WHO), Food and Agriculture Organization of the United Nations (FAO), and European Food Safety Authority (EFSA) were also considered where relevant. Search concepts covered nutrient bioavailability and bioaccessibility, food matrix effects, phytate, polyphenols, antinutritional factors, fermentation, germination, food fortification, biofortification, micronutrient status, dietary intake, and human nutritional outcomes. Search terms were combined using Boolean operators, synonyms, and database‐specific subject headings where available. Reference lists of relevant reviews and eligible primary studies were examined to identify additional publications. The search approach was informed by recommendations for transparent narrative review methods (Baethge et al. 2019).

#### 1.3.3 Eligibility Criteria and Study Selection

Eligible publications comprised primary experimental studies, randomized and non‐randomized human intervention studies, observational research relevant to nutrient intake or status, systematic reviews, meta‐analyses, and authoritative scientific guidance published within the specified date range. Studies were considered relevant when they investigated at least one determinant of nutrient supply or utilization, evaluated a nutritional intervention, or provided evidence needed to interpret nutritional outcomes. Publications were excluded when they lacked sufficient methodological information, were unrelated to the review objectives, or offered no substantive evidence concerning food properties, bioactive compounds, human factors, or nutritional strategies. Priority was given to peer‐reviewed research and reviews with clearly described methods and outcomes. Study selection was guided by relevance to the review questions and the need to distinguish experimental food‐processing evidence from evidence obtained directly in humans.

#### 1.3.4 Data Extraction and Critical Appraisal

Relevant information was organized according to study design, population or food matrix, target nutrient, proposed limiting mechanism, intervention characteristics, comparator, outcome measures, principal findings, and reported limitations. Particular attention was given to the distinction between nutrient concentration, nutrient retention after processing, in vitro bioaccessibility, human absorption, nutritional‐status biomarkers, and functional or clinical outcomes. This classification helped prevent mechanistic findings from being interpreted as proof of clinical benefit. The methodological strengths and limitations of individual publications were considered in relation to study design, sample size, duration, outcome validity, potential confounding, and applicability to real‐world dietary settings. SANRA principles were used to guide the clarity of the review rationale, literature‐search description, referencing, scientific reasoning, and presentation of relevant outcomes (Baethge et al. 2019).

#### 1.3.5 Synthesis and Interpretation

Evidence was synthesized thematically across four interconnected domains: food‐matrix and processing constraints; interactions involving bioactive and antinutritional compounds; human physiological and behavioral determinants; and intervention effectiveness and implementation (Baethge et al. 2019). Findings were compared according to their biological plausibility, consistency, methodological quality, and relevance to human nutrition. Contradictory findings and evidence gaps were retained in the synthesis rather than resolved through unsupported assumptions. No pooled effect estimate was calculated because the review was designed for critical narrative synthesis rather than quantitative meta‐analysis. Conclusions and recommendations were framed in proportion to the available evidence, distinguishing established human outcomes from promising mechanisms requiring further validation.

## 2 Factors Responsible for Low Nutrient Supply From Foods

### 2.1 Antinutrients

Antinutrients, also known as antinutritional factors, are natural/synthetic substances or compounds that inhibit nutrient absorption or availability (Figure 1). Nutritional studies mostly focus on antinutrients from food sources, including beverage sources. Antinutrients may act by binding to vitamins and minerals, preventing their uptake, or inhibiting enzymes (Carbas et al. 2020; Cominelli et al. 2022). Antinutrients be in form of drugs, proteins, chemical compounds naturally present in foods, or even excessive consumption of nutrients. They can prevent the uptake of nutrients such as minerals (Figure 1). Phytic acid is an example of antinutrients, and can strongly bind to minerals, for example, calcium, copper, zinc, magnesium, and iron, making them unavailable for absorption (Marolt et al. 2020). This causes precipitation that makes the minerals unavailable for intestinal absorption. Phytic acid is commonly found in the hulls of nuts, grains, seeds, etc., and is significant in animal nutrition, agriculture, eutrophication‐wise, and nutrient supply because of its mineral chelation properties and bound phosphates that are released in environment (Singh et al. 2015; Petroski and Minich 2020). Although milling involving hull removal can be used to detoxify or reduce phytate, however, phytic acids are commonly reduced/detoxified in feeds by the addition of “histidine acid phosphate type of phytases”. Oxalates and oxalic acid occur in several plants, and significantly occur in rhubarb, parsley, purslane, spinach, and tea (Marolt et al. 2020). Oxalates prevent absorption of calcium in the human by binding to it. Glucosinolates prevent iodine uptake, and affects thyroid functions, and are thus considered “goitrogens” (Lietzow 2021). They occur in plants like Brussels sprouts, broccoli, cabbage, radishes, mustard greens, cauliflower, etc.

**FIGURE 1:**
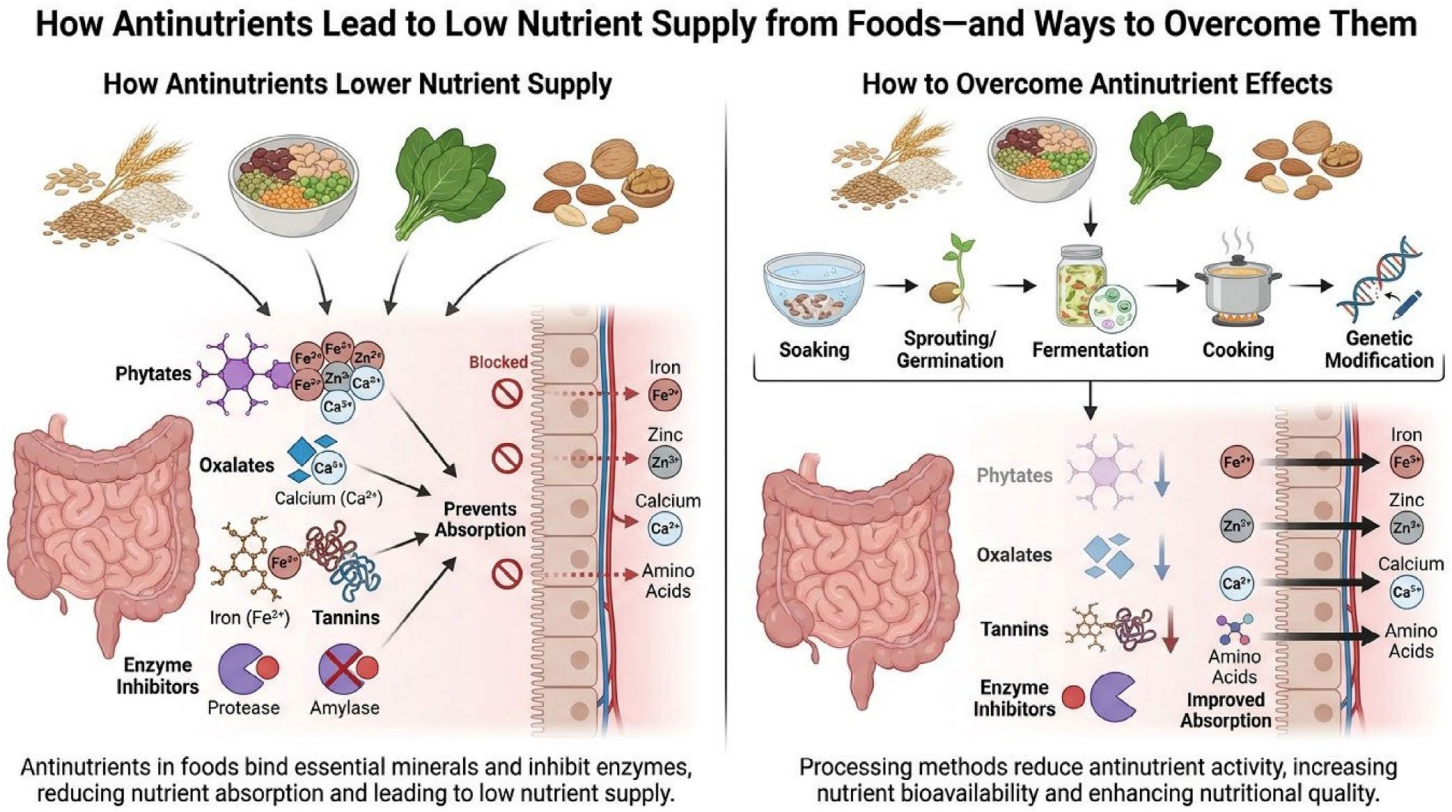
Antinutrients as barriers to nutrient bioavailability and strategies to improve nutrient utilization from foods. Antinutrients, including phytates, oxalates, tannins, and protease inhibitors, can reduce the bioavailability or digestibility of essential nutrients by binding minerals or interfering with protein digestion, potentially contributing to inadequate nutrient supply from foods. Appropriate food‐processing and preparation methods, such as soaking, germination, fermentation, and thorough cooking, can reduce the levels or activity of certain antinutrients and improve nutrient bioavailability and utilization. The effectiveness of these methods varies depending on the food matrix, antinutrient type, and processing conditions.

Some antinutrients can inhibit digestive enzymes, preventing nutrient supply from foods. Protease inhibitors are substances that inhibit the actions of trypsin, pepsin, and other proteases in the gut, preventing the digestion and subsequent absorption of protein (Kårlund et al. 2021). For example, Bowman–Birk trypsin inhibitor is found in soybeans (Kårlund et al. 2021). Some lectins and trypsin inhibitors in legumes interfere with nutrient digestion (Geraldo et al. 2022). Lipase inhibitors inhibit enzymes, including pancreatic lipase in humans that catalyze some lipids' hydrolysis. Intake of orlistat, an anti‐obesity drug, causes some fat to pass undigested via the digestive tract. Amylase inhibitor prevents the breakdown of glycosidic bonds of starches and other complex carbohydrates by interfering with the action of the enzymes that break them down, such as amylases, thus inhibiting simple sugars' release and absorption. This property has been explored in obesity treatment. Like lipase inhibitors, amylase inhibitors are employed as dietary aids for obesity treatment. They can be found in many beans. Commercial amylase inhibitors are also available and usually extracted from many sources, such as white kidney beans (Geraldo et al. 2022).

Other substances can also prevent nutrient supply from foods in the body. Excess consumption of nutrients can result anti‐nutritional actions. Excess consumption of dietary fiber can decrease intestinal transit time to such an extent that may interfere with the absorption of other nutrients, such as minerals (Carbas et al. 2020; Mekkara Nikarthil Sudhakaran and Bukkan 2021). However, this effect is not usually encountered in reality and reduced absorption of minerals is commonly attributed to the antinutrients such as phytic acids in foods with high dietary fiber. When high calcium containing foods are simultaneously consumed with iron containing foods, it can reduce iron absorption through an uncertain mechanism that involve iron transport protein hDMT1 that can be inhibited by calcium (Barbosa and Garcia‐Rojas 2022). Avidin found in raw egg whites is an antinutrient that tightly binds to vitamin B7 (Biotin) and can result in vitamin B7 deficiency in animals and humans, although in rare cases (Miranda et al. 2015). Polyphenolic compounds, including flavonoids, such as tannins, have widespread antinutrient properties. They chelate metals like zinc and iron, and reduce their absorption, and can inhibit digestive enzymes, as well as might precipitate proteins (Adamczyk et al. 2017). Saponins in many plants can act as antifeedants that can be considered antinutrients (Hussain et al. 2019; Melini and Melini 2021). Many antinutritional factors are significantly contributing to the problem of low nutrient supply from foods. Managing them is essential for the improvement of nutrient supply from dietary sources.

### 2.2 Effects of Food Processing

Food processing transforms agricultural produce into food, or transforms foods into other forms that may be more desirable and/or have longer shelf life. Food processing includes several ways of processing foods, from preliminary processing, such as grinding grains to make flour to street vendors that prepare ready‐to‐eat snacks to sophisticated industrial processing used to produce convenience foods to home cooking. Food processing can reduce the nutritional density of foods, and can even lead to complete destruction of some nutrients. The quantity of nutrient loss due to processing largely depends on the type of food, process parameters/conditions, and the method used (Vergeer et al. 2019; Starck et al. 2021). For example, the application of heat destroys some vitamins such as vitamin C (Better Health 2022). Consequently, canned fruits and other heat processed fruits and vegetables contain less vitamin C than fresh fruits and vegetables. Processing foods usually leads to nutrient losses, making it harder to meet nutritional needs of the body if the nutrients are not replaced through enrichment or fortification (Vergeer et al. 2019; Starck et al. 2021). In terms of food processing and nutrient loss, the question is often how to reduce the loss of nutrients. Refining/milling grains reduces their fiber, minerals, vitamins, etc., compared with whole grains (Kumar and Kalita 2017). Milling distributes some toxins and microorganism throughout the entire food inner and outer surface, which can cause the microorganisms to feed more on the nutrients, thus reducing nutrient density (Awuchi et al. 2020; Awuchi, Ondari, et al. 2021; Awuchi, Owuamanam, and Ogueke 2021). Consuming refined grains, for example, several processed foods, instead of whole grains can increase risks of diabetes, obesity, cancer, and high cholesterol (Tufail et al. 2021; Yasmin et al. 2021).

Certain vitamins have more stability and are less affected by the rigors of food processing than other vitamins. Vitamins (B‐group and C) soluble in water are more unstable compared to the vitamins (A, D, E, and K) soluble in fat, during storage and food processing. The most unstable vitamins are vitamin C, folate, thiamine, etc., while most stable vitamins are vitamin D, biotin, niacin, vitamin K, pantothenic acid, etc. (Better Health 2022). Many things take place during growing, storage, and harvesting that can also lead to the loss of vitamins. Milling cereals like wheat often involve removing the fibrous husks, which contain greater portion of the dietary fiber and other nutrients. This explains why food products like white bread have less nutrients compared to whole meal, even when they are artificially fortified with some lost nutrients (Kumar and Kalita 2017). It is practically very unlikely to add all that were lost, especially the phytochemicals (Awuchi, Atoki, and Echeta 2026; Awuchi, Iyiola, et al. 2026). The ‘fiber’ used to replace lost nutrients in some products is usually resistant starch, which might have equivalent benefits as the lost fiber. Boiling foods such as potato can lead to migration of water‐soluble vitamins into the boiling water (Better Health 2022), although one may still benefit from the leached nutrients when the liquid is consumed. Some food processing methods such as steaming, stir‐frying, grilling, roasting, microwaving, irradiation, etc., can also lead to the loss of one nutrient or the other.

### 2.3 Postharvest Storage and Loss

After harvest, foods undergo physical, enzymatic, and microbial changes that can affect weight, quality, safety, and selected nutrients (Food and Agriculture Organization of the United Nations [FAO] 2025; Awuchi 2026b). The magnitude and speed of change vary substantially with commodity, maturity, temperature, humidity, light, packaging, handling, and storage duration; therefore, a single estimate such as “30% of nutrients are lost after three days” should not be generalized across produce or nutrients without a defined source and measurement method. Food loss estimates are also not identical to nutrient‐loss estimates: mass lost, edible portions discarded, nutrient degradation, and changes in bioavailability are distinct outcomes. FAO's Food Loss Index shows substantial commodity and regional variation, with fruits and vegetables generally more perishable than cereals and pulses (FAO 2025). Figure 2 shows the annual nutritional postharvest loss using maize ( *Zea mays* ) as a case study.

**FIGURE 2:**
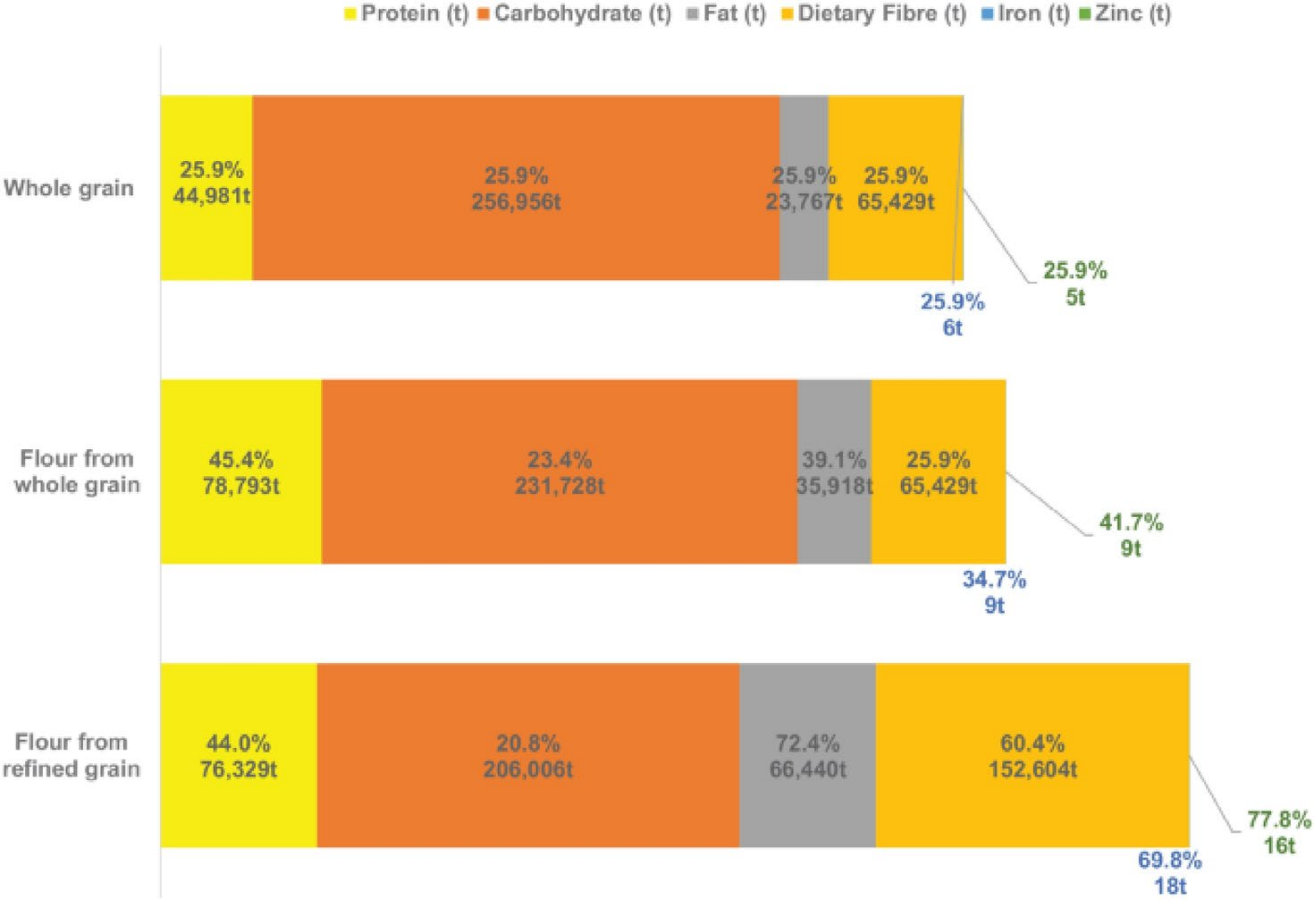
Predicted annual nutritional postharvest loss: A case study of maize ( *Zea mays* ). Adapted from Bechoff et al. (2022).

Storage plays a vital role in the food supply chain, and several studies reported that maximum losses happen during this operation (Majumder et al. 2016; Awuchi 2026b). There is seasonal cultivation of crops in most places. After harvest, foods such as grains are stored as food reserves for a short or prolonged period, and are also reserved as seeds for the subsequent farming season. Storage is indispensable in the food value chain. In developing nations like India, around 50% to 60% of grains are traditionally stored in local structures, such as Sanduka, earthen pots, Kanaja, Kothi, Kacheri, and Gummi, at the farm and household level for seed purpose and for self‐consumption, and the same traditional storage is reported in underdeveloped countries, such as Burundi, Central African Republic, Bangladesh, etc. (Kumar and Kalita 2017; Strecker et al. 2022). Cereal grains and tubers form the major staple foods in developing countries, and are responsible for the major postharvest loss on the basis of calorific loss of all agricultural produce. Sadly, around 50% to 60% of cereal grains are lost during storage mainly due to lack of technical know‐how (Kumar and Kalita 2017). Employing scientific methods of storage, such as atmospheric storage, silos, preservative measures, etc., can reduce this postharvest loss to 1%–2% or less. Reducing postharvest losses is critical to improving nutrient supply from foods.

### 2.4 Presence of Microorganisms

Microorganisms influence nutrient supply through at least four distinct pathways. First, spoilage organisms can degrade food and reduce the edible quantity available. Second, pathogens create health risks and may make food unsafe even when its nutrient composition is largely unchanged. Third, toxigenic fungi can contaminate commodities with mycotoxins, including aflatoxins in susceptible crops such as maize and groundnuts (Awuchi 2026a, 2026c); these hazards require prevention, testing, and safe supply‐chain controls rather than relying on visible spoilage alone (Partnership for Aflatoxins Control in Africa 2018; Awuchi et al. 2020; Awuchi, Ondari, et al. 2021; Awuchi, Owuamanam, and Ogueke 2021). Fourth, selected bacteria, yeasts, and molds are deliberately used in fermentation, where their enzymes may improve digestibility, modify flavor, and reduce some antinutritional compounds. Fermentation is not uniformly beneficial, however: outcomes depend on the organism, substrate, process control, and food safety conditions. Microbial activity should therefore be evaluated as a context‐dependent process, not simply as nutrient depletion (Kumar and Kweera 2013; Awuchi et al. 2020; Awuchi, Ondari, et al. 2021; Awuchi, Owuamanam, and Ogueke 2021; Awuchi, Nwozo, et al. 2022; Awuchi, Ondari, et al. 2022).

### 2.5 Poor Bioavailability of Nutrients

Nutrient bioaccessibility is the fraction released from the food matrix during digestion and potentially available for intestinal uptake; bioavailability refers more broadly to the fraction absorbed and available for physiological use. These concepts should not be used interchangeably. In vitro digestion models can compare release under standardized conditions, but cannot independently establish human absorption or clinical benefit. Absorption studies, biomarkers, functional outcomes, and controlled trials answer different questions and should be interpreted accordingly (Melse‐Boonstra 2020; Chouraqui 2022). For example, nonhaem iron absorption is generally inhibited by phytate and some polyphenols and enhanced by ascorbic acid, but the size of the effect depends on meal composition, iron status, and the study method. A single food constituent should not be assumed to determine the nutritional value of an entire diet. Poor bioavailability of nutrients can contribute to their poor supply and uptake from foods. Bioavailability describes the effects of metabolic factors on the utilization of nutrients. Nutrients supply from food to the body does not only depend on the amount of nutrients in foods, but also depend on their bioavailability (Melse‐Boonstra 2020). Nutrient bioavailability highly varies, and can be affected by many factors. Different nutrients, such as protein, vitamins, minerals, fats, etc., and the forms they exist in the medium of ingestion, will react differently to enhancers, inhibitors, and the nutritional status of the hosts, all of which influence the bioavailability of the nutrient (Melse‐Boonstra 2020). Antinutrients are common factors that slow the bioavailability of nutrients. Dietary fibers found in plant foods slow down nutrient absorption. Phytochemicals such as polyphenols are another class of inhibitors of nutrient bioavailability. The food microstructure and matrix affect the supply of nutrients from foods by affecting their availability. Nutrient bioavailability is also influenced by many other factors including diet, nutrient concentration, nutritional status, health, and life‐stage (Chouraqui 2022). Consequently, improving the bioavailability of nutrients in a population will improve their nutritional status by improving the way their body utilizes nutrients from foods (Fan et al. 2019).

### 2.6 Human and Genetic Factors, Including Intolerance (e.g., Lactose Intolerance)

The low nutrient supply from foods can also be explained by intrinsic factors in the human body (see Table 2), ranging from factors that reduce the bioavailability of nutrients to disease conditions and genetic factors (Figure 3) that impair nutrient utilization in the body (Diana et al. 2017; Hodge and Taylor 2022). Vitamin A deficiency (VAD) and iron deficiency (ID) or zinc deficiency (ZD) usually occur at the same time due to dietary patterns and many social factors, and also due to likely interactions among micronutrients or their regulation, and can be affected by the host's status in regard to disease and infection (Suzuki and Tomita 2022). Some nutritional studies focused on exploring the connection between iron status and vitamin A intake. Vitamin A supplementation has effects on hemoglobin concentrations in the blood (Diana et al. 2017; Hodge and Taylor 2022). VAD is associated with the metabolism of iron and its reduced incorporation into erythrocytes. Human studies have associated the transcription of several hepatic genes and erythropoietin synthesis. The status of vitamin A may have an influence on the catabolism and synthesis of proteins involved in hepatic iron mobilization and storage. Htet et al. (2014) showed that iron status and vitamin A are interdependent. This was further confirmed by da Cunha et al. (2019), who conducted a meta‐analysis and systematic review on the influence of supplementation of vitamin A on iron status. Htet et al. (2014) and da Cunha et al. (2019) reported that improved iron status is connected with higher serum retinol, and concluded that improved vitamin A is required to decrease ID. It shows that using vitamin A and iron supplements simultaneously is more effective for the prevention of ID than using only one micronutrient. Conversely, there is not enough knowledge of iron effects on vitamin A status and metabolism, although some studies have tried providing knowledge in this area. It appears that ID affects vitamin A by impairing the mobilization of vitamin A stores in the liver, resulting in low serum retinol and increased hepatic retinoids (Borel et al. 2015). This was attributed to reduced retinyl ester hydrolases. Studies have described possible interactions between iron and zinc, where combined iron and zinc supplementation showed reduced effectiveness compared with supplementation with only iron or zinc. This is because zinc and iron have similar absorption pathways, which may cause competition between these two minerals during their absorption (Kondaiah et al. 2019; Zhang, Liu, et al. 2022). Some disease conditions can prevent, or at least impair, nutrient absorption and utilization in the body. Some diseases such as soy milk protein intolerance, celiac disease, biliary atresia, lactose intolerance, chronic pancreatitis, cystic fibrosis, Shwachman‐Diamond syndrome, Whipple disease, cow's milk protein intolerance, digestive disorders, etc. have been shown to reduce/impair nutrient uptake from foods (Montoro‐Huguet et al. 2021a, 2021b; Khalid et al. 2022).

**TABLE 2:** Human factors leading to low nutrient absorption and corrective nutritional interventions.

Human factor category	Specific factor	Nutrient affected	Contribution to low supply	Mechanism of impairment	Impact on nutrient uptake	Associated food interaction	Related bioactive component	Nutritional measure	Implementation approach	Anticipated outcome (References)
Physiological	Aging	Vitamin B12	Reduced intrinsic factor	Impaired cobalamin binding	Intrinsic‐factor loss can markedly reduce active B12 absorption	Meat and dairy	None directly	Intramuscular supplementation	Monthly injections	Normalized status in elderly (Richards et al. 2025)
Pathological	Celiac disease	Folate	Villous atrophy	Damaged absorptive surface	Malabsorption can occur with villous injury; magnitude varies	Wheat products	Gluten peptides	Gluten‐free diet adoption	Alternative grains	Mucosal recovery, absorption restored (Montoro‐Huguet et al. 2021a, 2021b)
Lifestyle	Medication use (antacids)	Iron	Altered gastric pH	Nonheme iron insolubility	Potentially reduced; effect depends on dose and meal composition	Plant sources	Polyphenols worsening	Ascorbic acid co‐ingestion	Citrus‐enhanced meals	pH‐independent uptake improved (Nicholas et al. 2026)
Genetic	MTHFR polymorphism	Folate	Impaired methylation	Reduced active form conversion	Effect varies by genotype and folate status	Leafy greens	None	Methylfolate supplementation	Targeted nutraceuticals	Bypassed defect, levels optimized (Dötsch‐Klerk et al. 2023)
Environmental	Stress	Magnesium	Cortisol‐mediated excretion	Increased urinary loss	Evidence and prevalence are context‐dependent	Nuts	Saponins interacting	Magnesium‐rich relaxation aids	Herbal teas with Mg	Homeostasis restored (Fanzo et al. 2023)
Developmental	Pregnancy	Calcium	Increased fetal demand	Competitive maternal‐fetal transfer	Maternal depletion risk	Dairy	Caseins aiding	Fortified prenatal supplements	Daily regimens	Bone health preserved (Defraeye et al. 2025)
Microbial	Dysbiosis	Vitamin K	Altered bacterial synthesis	Reduced menaquinone production	Coagulation issues	Fermented foods	Probiotics restoring	Probiotic yogurt consumption	Daily fermented intake	Microbiome balanced, synthesis enhanced (Bassaganya‐Riera et al. 2021)
Socioeconomic	Food insecurity	Multiple micronutrients	Limited access	Inadequate intake variety	Widespread deficiencies	Staples only	None	Community fortification programs	Staple food enrichment	Population‐level improvements (Levis et al. 2024)

**FIGURE 3:**
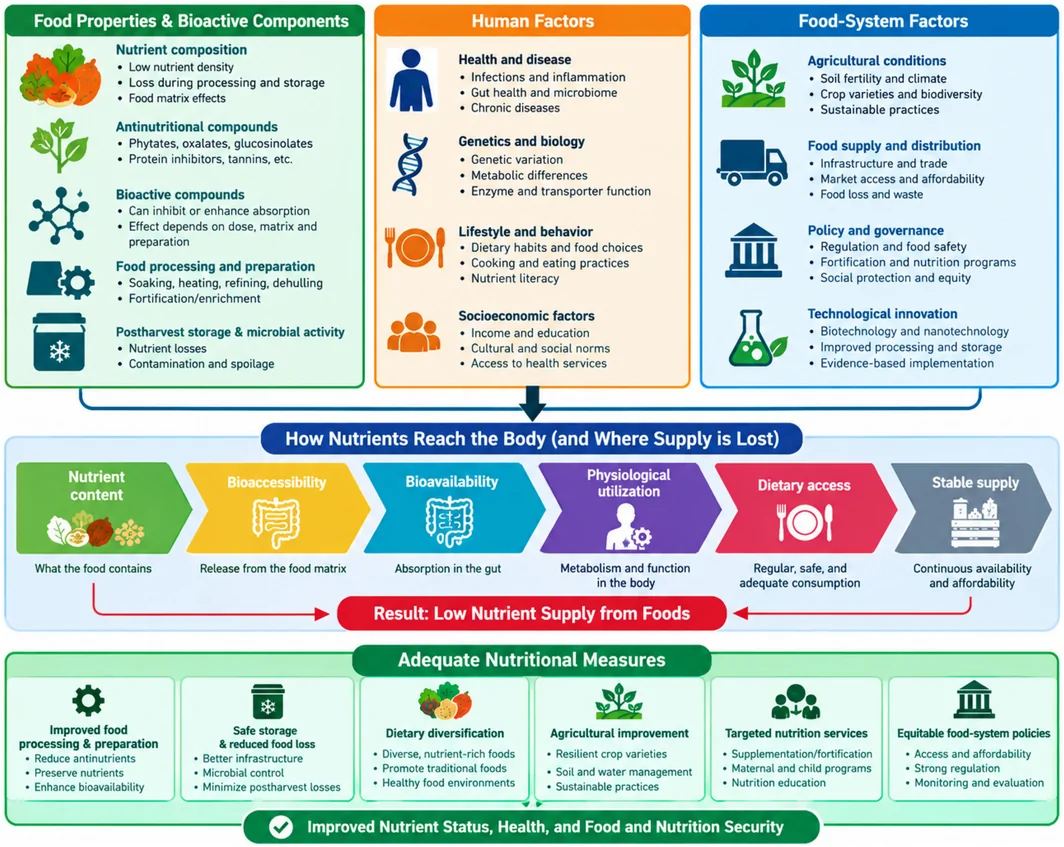
Food properties, bioactive components, and human factors contributing to low nutrient supply: Potential for improvement through nutritional measures. Food nutrient composition, antinutritional and bioactive compounds, processing, storage, human physiological and genetic factors, and food‐system conditions influence nutrient bioaccessibility, absorption, utilization, dietary access, and supply stability. Appropriate nutritional measures, including improved food processing, safe storage, dietary diversification, agricultural improvement, targeted nutrition services, and equitable food system policies, can address these barriers and support improved nutrient status, health, and food and nutrition security.

Genetic variation is a key factor to explain the huge interpersonal variability in the bioconversion and bioavailability of provitamin A carotenoids into vitamin A (Suzuki and Tomita 2022). β‐carotene 15,15′‐monooxygenase (BCMO1) is responsible for BC conversion into retinal. Genetic polymorphisms that contribute to the poor converter phenotype have been described (Suzuki and Tomita 2022). Many single nucleotide polymorphisms (SNPs) in the BCMO1 coding region have been identified and show that the enzyme's catalytic activity may be reduced by 48% to 59%. Borel et al. (2015) reported that in 12 genes, 25 SNPs explained 69% of variation in the response of post‐prandial chylomicron β‐carotene. Of all the genes, the BCMO1 (BCO1) and the ABC transporter family were indirectly or directly effectively associated with the response of the post‐prandial chylomicron β‐carotene (Borel et al. 2015). This shows that genetic variation has to be integrated into studies as it can have an effect on biofortified crops' effectiveness to improve micronutrient supply in many populations, especially in developing countries.

### 2.7 Poor Agricultural Practices

Agricultural conditions influence both the quantity and composition of food. Soil organic matter, pH, water availability, nutrient balance, crop variety, and agronomic management can alter crop yield and concentrations of selected minerals and phytochemicals. Climate change compounds these pressures through heat stress, drought, flooding, changing pest burdens, and disruption of storage and distribution. However, “sustainable” or “organic” production should not automatically be equated with greater nutrient density: crop species, cultivar, soil, fertilization, maturity, and analytical methods vary, and evidence differs by nutrient and setting. Priorities include soil testing and balanced fertilization, crop diversification, conservation practices, irrigation efficiency, climate‐resilient varieties, and evaluation of nutrient outcomes alongside yield, affordability, and environmental impact (López‐Yerena et al. 2019; Montgomery and Biklé 2021). Poor farming practices are shortcuts that destroy their environment to the point that the environment can no longer sustain normal, healthy growth of crops with the required levels of nutrients. Examples include using excessive chemicals, over‐tilling, and monocropping. Made‐made soil erosion inhibits our ability to grow nutritious food. By decreasing the nutrients available to plants as well as the space for them to put down roots, soil erosion can decrease crop yields by up to 50% (Montgomery and Biklé 2021). In addition, crops that do grow tend to be of a lower quality: misshapen, smaller and less nutritious (Konecny et al. 2019). Wastage of foods during and after harvest increases the prices of food to consumers and could reduce food security in a country. Up to 1/3 of all food is lost or wasted worldwide. The most wasted foods are those the people need the most. Table 3 summarizes the food properties contributing to reduced nutrient bioavailability and strategies for enhancement, as well as the bioactive components influencing nutrient supply and mitigation approaches.

**TABLE 3:** Food properties contributing to reduced nutrient bioavailability and strategies for enhancement (and bioactive components influencing nutrient supply and mitigation approaches).

Food category	Specific example	Nutrient affected	Property causing low supply	Mechanism of action	Impact on absorption	Associated bioactive component	Interacting human factor	Nutritional measure	Implementation approach	Anticipated outcome (References)
Cereals	Wheat bran	Iron	High phytate content	Chelation of minerals forming insoluble complexes	Inhibits non‐haem iron absorption; magnitude varies	Polyphenols enhancing binding	Aging‐related reduced gastric acid	Phytase enzyme addition during processing	Fermentation or supplementation in flour	May improve iron bioaccessibility/absorption; verify human evidence (Richards et al. 2025)
Legumes	Soybeans	Zinc	Fiber entrapment	Physical binding within plant matrix	May reduce zinc bioavailability; magnitude varies	Tannins inhibiting transporters	Pregnancy‐induced increased demand	Germination and soaking pre‐consumption	Home‐based processing techniques	May improve zinc bioavailability; verify human evidence (Reboul et al. 2026)
Vegetables	Spinach	Calcium	Oxalate presence	Formation of calcium oxalate crystals	Calcium bioavailability can be limited by oxalate; human effect varies	Flavonoids modulating solubility	Gastrointestinal dysbiosis	Pairing with vitamin D‐rich foods	Meal composition adjustment	Potential improvement; avoid quantifying without direct evidence (Weaver et al. 2025)
Fruits	Tomatoes	Lycopene	Cellular matrix rigidity	Entrapment in chromoplasts	Limited release during digestion	Carotenoids requiring lipid co‐ingestion	Medication interference (e.g., antacids)	Thermal processing like cooking	Incorporation in sauces or stews	Doubled serum levels post‐intervention (Jia et al. 2024)
Dairy	Unfermented milk	Magnesium	Casein phosphorylation	Competitive binding at absorption sites	Uptake reduced by 25%	Lactose facilitating but incomplete	Lactose intolerance	Fermentation to yogurt	Probiotic‐enriched variants	Absorption enhanced by 35% (Melse‐Boonstra et al. 2020)
Nuts	Almonds	Vitamin E	Lipid oxidation susceptibility	Degradation during storage	Potentially reduced; effect depends on dose and meal composition over time	Tocopherols self‐protecting minimally	Obesity‐linked inflammation	Vacuum packaging and antioxidant addition	Industrial fortification	Sustained levels with 80% retention (Wuni et al. 2025)
Grains	Rice	Folate	Starch granule encapsulation	Hindered enzymatic release	Absorption at 30%–40% efficiency	Glucosinolates indirectly affecting	Genetic polymorphisms in folate metabolism	Biofortification via breeding	Genetically enhanced varieties	Uptake improved to 60% (Nicholas et al. 2026)
Tubers	Potatoes	Potassium	Anti‐nutritional saponins	Membrane disruption limiting transport	Reduced cellular availability	Alkaloids exacerbating	Chronic kidney disease	Peeling and boiling	Minimal processing methods	Bioavailability restored to near 90% (Bhardwaj et al. 2024)

Bioactive category	Specific component	Nutrient influenced	Role in low supply	Mechanism of influence	Impact on nutrient delivery	Associated food source	Interacting human factor	Nutritional measure	Implementation approach	Anticipated outcome (References)
Polyphenols	Flavonoids	Iron	Antagonistic binding	Inhibition of nonheme iron absorption	Reduction by 50%–70% in plant‐based diets	Tea and coffee	Menstrual blood loss	Timing separation from meals	Consume 2 h apart	Minimized interference, absorption up by 40% (Arshad et al. 2025)
Carotenoids	Beta‐carotene	Vitamin A	Low solubility	Poor micellar incorporation	Bioavailability at 10%–30%	Carrots	Gut microbiota imbalance	Co‐consumption with fats	Addition of oils in cooking	Conversion efficiency doubled (Machado et al. 2025)
Glucosinolates	Isothiocyanates	Selenium	Competitive uptake	Interference with selenoprotein synthesis	Decreased status in cruciferous‐heavy diets	Broccoli	Thyroid dysfunction	Balanced intake with selenium sources	Mixed vegetable diets	Restored selenium levels (Reboul et al. 2026)
Alkaloids	Caffeine	Calcium	Increased excretion	Renal calcium loss stimulation	Net loss of 5–10 mg per cup	Coffee	Postmenopausal bone fragility	Calcium fortification in beverages	Supplemented alternatives	Compensated loss, bone density maintained (Chandran et al. 2024)
Saponins	Glycosides	Protein	Digestive enzyme inhibition	Reduced proteolysis	Amino acid availability lowered by 20%	Quinoa	Elderly protein malabsorption	Rinsing and heat treatment	Pre‐cooking preparation	Digestibility improved to 85% (Cui et al. 2026)
Oligosaccharides	Fructans	Magnesium	Fermentation byproducts	Osmotic effects reducing absorption	Uptake decreased in sensitive individuals	Onions	IBS‐related malabsorption	Gradual dietary introduction	Incremental portions	Tolerance built, absorption normalized (Lu et al. 2025)
Phenolic acids	Chlorogenic acid	Zinc	Chelation in gut lumen	Formation of stable complexes	Bioavailability reduced to 40%	Apples	Pediatric growth demands	Zinc supplementation pairing	Fortified snacks	Compensatory increase in status (Gonçalves et al. 2026)
Terpenes	Limonene	Folate	Oxidative stress induction	Folate degradation acceleration	Lower serum levels in citrus‐rich diets	Lemons	Genetic MTHFR variants	Antioxidant co‐administration	Vitamin C‐enriched formulations	Stability enhanced, levels sustained (Riccardi et al. 2022)

## 3 Food and Nutrition Insecurity

Food and nutrition insecurity concerns whether people can reliably obtain sufficient, safe, and nutritionally adequate foods. Its consequences arise through multiple pathways: low purchasing power can restrict dietary diversity; weak infrastructure can reduce availability and increase losses; unsafe water and food can increase infection; and illness can impair intake, absorption, or utilization. These mechanisms can reinforce one another, but food insecurity should not be treated as a direct proxy for a specific micronutrient deficiency. Assessment should combine food‐access measures with dietary diversity, nutrient intake, biomarkers where appropriate, and health‐system context (FAO et al. 2019, 2020; Brown et al. 2019; Pereira et al. 2022). Food and nutrition insecurity is described as “limited or uncertain availability of nutritionally adequate and safe foods or limited or uncertain ability to acquire acceptable foods in socially acceptable ways” (FAO et al. 2019; Brown et al. 2019; Pereira et al. 2022). Individuals whose food intakes reduce below their minimum requirements of calories are considered food insecure, along with those who are physically unfit, or have any disease associated with hunger, lack of food, or unbalance diet (Ahmad et al. 2021). Food insecurity can also be defined as “Food insecurity exists when people do not have adequate physical, social or economic access to the sufficient, safe and nutritious food to meet their dietary needs and food preferences for an active and healthy life” (FAO et al. 2019; Brown et al. 2019; Pereira et al. 2022). According to FAO Food Insecurity is defined as “a situation that exists when people lack secure access to sufficient amounts of safe and nutritious food for normal growth and development and active and healthy life” (FAO et al. 2019). No matter what the description may be, the fact is that food and nutrition insecurity affect the supply of nutrients from food. Food and nutrition insecurity limits food nutrients from reaching to the affected population.

## 4 Strategies and Policies to Improve Nutrient Supply From Food at National and International Levels: Current and Future Recommendations

### 4.1 Food Fortification/Enrichment

Food fortification is a proven, safe, and cost‐effective strategy for improving diets and for the prevention and control of micronutrient deficiencies. Food fortification/enrichment can help improve nutrient supply from foods. Food enrichment/fortification is the addition of micronutrients to foods. The staple diets in a region can be deficient or contain insufficient micronutrients because of the inherent deficiencies in the staple food matrix or due to the local soil; adding micronutrients to condiments, staples, and/or suitable delivery vehicles can prevent large‐scale deficiency diseases in affected populations (Olson et al. 2021).

Many food products have been fortified worldwide, whether by voluntary action or by law. Although some view fortification/enrichment as a marketing strategy, a lot of work must go into food products before simply enriching/fortifying them. To carry out fortification/enrichment, it must first be established that the micronutrient addition is beneficial to health, is safe, and the food delivery vehicle will be effective (Olson et al. 2021). These additions must also obey all food policies and labeling regulations, and support nutritional science.

Vitamin fortification programs already exist in at least one country for vitamin D, riboflavin, vitamin B_6_, thiamine, folate, niacin, vitamin A, vitamin B_12_, and vitamin E. Mineral fortification program captures iodine, iron, calcium, fluoride, zinc, selenium, etc. Currently, more than 140 countries require food fortification with one or more nutrients (Global Fortification Data Exchange 2022). Folate is the most fortified vitamin in over 62 countries, while wheat flour (enriched flour) is the most fortified food (Global Fortification Data Exchange 2022). This and other products can be harnessed to improve nutrient supply from foods.

Examples of foods and beverages that have been fortified are presented in this paragraph. Iodised salt has been used since World War II to caution iodine deficiency. Folate (folic acid) reduces blood homocysteine levels, improves proper growth and cell division, helps form red blood cells, and prevents neural tube defects (NTDs). Adding folic acid to flour prevents many cases of NTDs in infants. Niacin can be added to bread to reduce the incidence of pellagra, commonly seen in populations that use corn and corn products as their main diet (Olson et al. 2021; Global Fortification Data Exchange 2022). Although corn contains niacin, the niacin in corn is not bioavailable without undergoing nixtamalization (alkali treatment), and as a result, it does not contribute to the overall niacin intake. Fluoride prevents tooth decay and maintains dental health and can be fortified using water as the vehicle (Sabti et al. 2019; Dey et al. 2022). Excess should be avoided to prevent fluorosis (condition due to excess intake of fluoride).

### 4.2 Social Safety Net (SSN)

SSN consists of noncontributory assistance that exist to better lives of individuals and vulnerable families experiencing destitution and poverty (World Bank 2018). Low and stagnating incomes among the poor has meant that low purchasing power remains a serious constraint to household food and nutritional security (Fares and Puig‐Junoy 2021; Miller‐Graff et al. 2021). A case study can be drawn from SSN practice in Egypt. In Egypt, food subsidies are as old as the pyramids, and recently receiving structural reforms to improve supply of foods and nutrients to vulnerable individuals and households (Alderman 2019). It combined rationed commodity and bread subsidies support before 2014 reforms were only at the margin, partly determined by deficits and inflation, but everyone agrees a social contract have to exist to make food affordable and available (Alderman 2019; Fares and Puig‐Junoy 2021; Miller‐Graff et al. 2021). A targeted transfer of cash runs along with the food‐oriented program, although currently overshadowed by the latter. In general, SSN has been shown to be effective in improving nutrient supply from foods to vulnerable households.

### 4.3 Improved Food Processing and Production

Food processing can be done in a way that will minimize nutrient loss or even improve the nutritional value of the processed foods (Figure 4). Preserving the nutrient value of foods such as vegetables, grains, tubers, fruits, etc., during processing is very possible. Some suggestions to retain the maximum nutrition in the foods you cook include: storing foods properly, such as keeping cold foods cold and sealing some foods in airtight containers; keeping vegetables in the crisper section of the refrigerator; washing or scrubbing vegetables rather than peeling them; using the outer leaves of vegetables like cabbage or lettuce unless they are wilted or unpalatable; microwave, steam, roast or grill vegetables rather than boiling them; if you boil your vegetables, save the nutrient‐laden water for soup stock; use fresh ingredients whenever possible; cook foods quickly; etc. (Kumar and Kalita 2017; Better Health 2022; Awuchi 2026c). Food processing methods that destroy essential nutrients should be avoided (Awuchi 2026c). Many traditional methods of food preparation such as germination, fermentation, cooking, and malting increase the nutritive quality of plant foods through reducing certain antinutrients such as phytic acid, polyphenols, and oxalic acid (Marolt et al. 2020; Kårlund et al. 2021; Geraldo et al. 2022). Such processing methods are widely used in societies where cereals and legumes form a major part of the diet. An important example of such processing is the fermentation of cassava to produce cassava flour: this fermentation reduces the levels of both toxins and antinutrients in the tuber.

**FIGURE 4:**
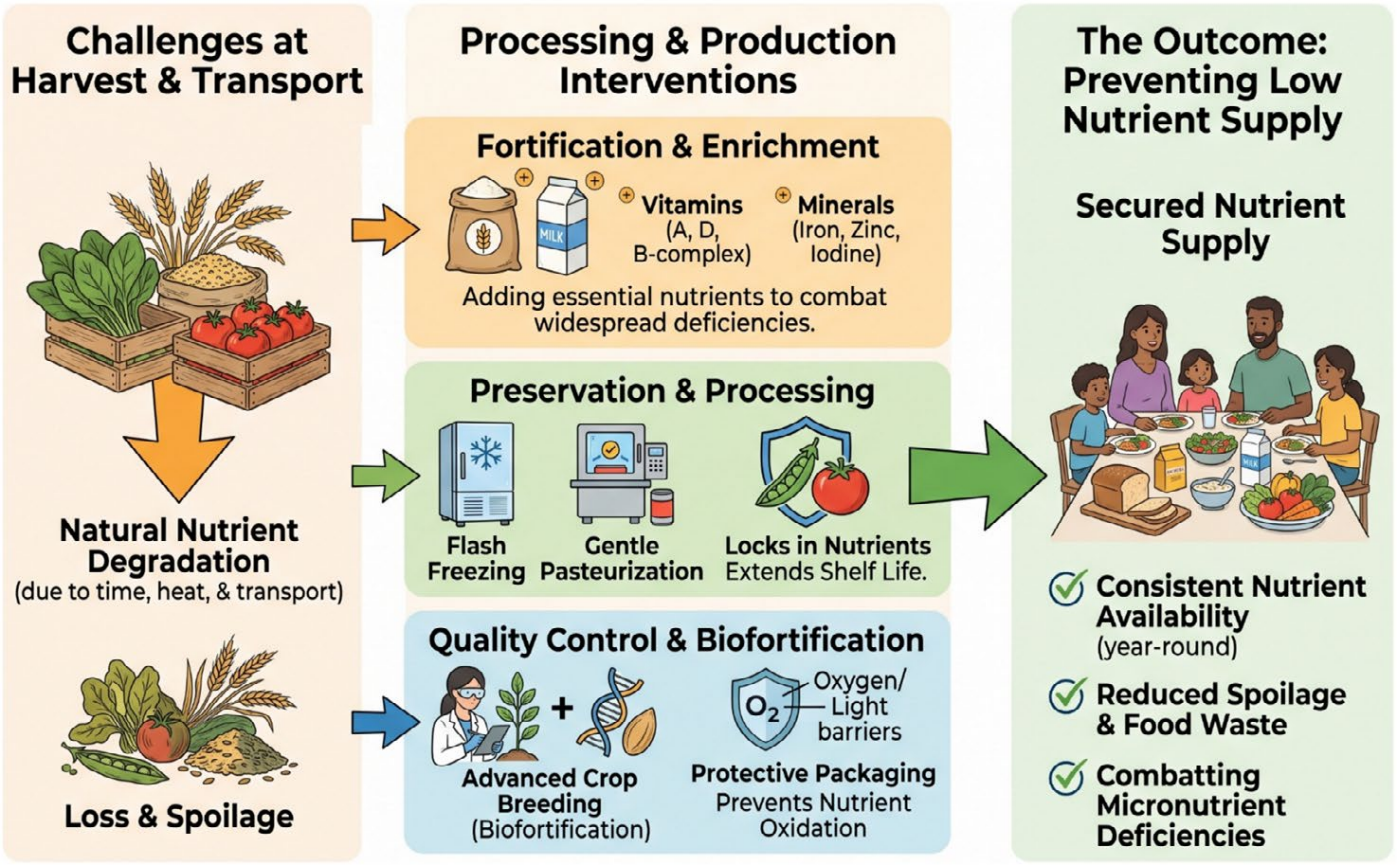
Improved food processing and production to prevent inadequate nutrient supply from foods. Improved food processing and production practices help prevent inadequate nutrient supply by enhancing the nutritional quality, bioavailability, and retention of essential nutrients in foods. These practices also help reduce nutrient losses during processing, storage, and distribution, thereby improving food quality and supporting adequate dietary nutrient intake.

### 4.4 Food and Nutrition Security

Food and nutrition security is not secured by aggregate supply alone. It requires physical and economic access to safe, diverse foods, adequate utilization, and stability over time. A useful policy analysis identifies which pillar is limiting in a particular setting and examines how gender, age, disability, conflict, market concentration, food prices, and seasonal variation shape that constraint. The Sustainable Development Goals provide a shared policy framework, but progress should be measured through diet quality and nutritional outcomes as well as production or calorie availability (FAO et al. 2020; FAO 2021). Food and nutrition security is the measure of the availability of food and individuals' ability to access it to meet their dietary needs. The international and local communities should ensure there is food and nutrition security by making sure that all people, at all times, have physical, social, and economic access to sufficient, safe, and nutritious food that meets their food preferences and dietary needs for an active and healthy life. The availability and accessibility of nutritious food irrespective of class, gender or region is another one is very important to ensure adequate supply of nutrients from foods (Jellason et al. 2021). Similarly, household food security is very important and can be considered to exist when all members of all the households, at all times, have access to enough food for an active, healthy, and robust life. Individuals who are food secure do not live in hunger or fear of starvation (FAO et al. 2020). Multiple different international agreements and mechanisms have been developed to address food security. The main global policy to reduce hunger and poverty is in the Sustainable Development Goals. These goals should be strictly targeted to meet the growing need for food security worldwide, especially in developing countries. In particular Goal 2: Zero Hunger sets globally agreed on targets to end hunger, achieve food security and improved nutrition, and promote sustainable agriculture by 2030, and can be directed to ensure adequate supply of nutrients to all households (FAO et al. 2020). The Food and Agriculture Organization of the United Nations, or FAO, identified the four pillars of food security as availability, access, utilization, and stability. The WHO states that there are three pillars that determine food security: food availability, food access, and food use and misuse. The FAO adds a fourth pillar: the stability of the first three dimensions of food security over time.

#### 4.4.1 Availability

Food availability relates to the supply of food through production, distribution, and exchange. As food consumers are more than producers, food must be made available through adequate distribution to all regions/nations. Food chain infrastructure and storage technologies on farms can also affect the amount of food wasted in the distribution process (FAO et al. 2020). Food exchange requires efficient market institutions and trading systems to food and nutrition security (FAO 2021).

#### 4.4.2 Access

Food and nutrition insecurity is best understood as a structural constraint on dietary adequacy, not merely as a shortage of calories. Availability, economic and physical access, utilization, and stability interact: a market may contain nutrient‐rich foods that households cannot afford, while households with sufficient food expenditure may still consume monotonous diets or face infection, unsafe water, and barriers to healthcare. Policy analysis should therefore assess diet quality, affordability, intrahousehold distribution, seasonal variation, and health service access alongside food quantity. The four‐pillar framework is useful, but listing its components without identifying the binding local constraint does not establish which intervention is likely to work (FAO et al. 2019, 2020; Jellason et al. 2021). It is noteworthy that hunger and malnutrition can be mainly caused not just by food scarcity but by inability to have access to available foods, mostly caused by poverty. Access depends on whether the household has enough income to purchase food at prevailing prices or has sufficient land and other resources to grow its own food (Jellason et al. 2021; FAO 2021). Direct access and economic access have to be ensured in our communities and supported by government and interest groups. Access to foods have to be made available in socially acceptable manner, without waiting for emergency food supplies, stealing, scavenging, or other coping strategies (FAO 2021).

#### 4.4.3 Stability

Food stability should be taken seriously. Factors responsible for food instability should be avoided at all costs. Drought, flooding, and natural disasters should be avoided as they often result in decreased food availability and crop failure and can even wipe out entire food supply chain (Rasul et al. 2018). The developing and underdeveloped countries are mostly affected by food instability. Civil conflicts can also decrease access to food. Instability in markets resulting in food price spikes can cause transitory food insecurity (FAO 2021). Loss of employment and productivity are other factors that can temporarily cause food insecurity. Food should be stable to improve nutrient supply from dietary sources.

#### 4.4.4 Utilization

Utilization is another important factor. To attain food security, ingested foods must be safe and enough to meet individuals' physiological requirements (Rasul et al. 2018). Food safety affects food utilization, and can be affected by the preparation, processing, and cooking of food in the community and household. Access to healthcare is another determinant of food utilization, since the health of individuals controls how the food is metabolized (FAO et al. 2020).

### 4.5 Nanotechnology (Nanoencapsulation)

Nanoencapsulation is an emerging food‐delivery strategy in which nutrients or bioactive compounds are enclosed within nanoscale carriers to improve stability, dispersibility, protection against degradation, and potentially release during digestion. Encapsulation may be particularly useful for oxidation‐sensitive vitamins, poorly water‐dispersible micronutrients, and compounds whose efficacy is constrained by interactions with the food matrix. At national and international levels, it could complement conventional fortification and biofortification by improving nutrient retention during processing, storage, and gastrointestinal transit. However, enhanced delivery should not be equated with proven improvements in human nutritional status; clinical benefits must be established for each nutrient, formulation, and target population (McClements 2020). However, national and international implementation requires stronger evidence on safety, exposure, and feasibility. Characterization should establish particle‐size distribution, composition, surface properties, encapsulation efficiency, stability, and dissolution or degradation under food‐processing and gastrointestinal conditions. Exposure assessment must estimate intake across population groups, including high consumers and potentially vulnerable individuals. Toxicological evaluation should consider gastrointestinal effects, systemic absorption, genotoxicity, immune responses, microbiome interactions, and possible accumulation where relevant. In the European Union, EFSA guidance requires nanospecific characterization and risk assessment when applicable; regulatory requirements differ across jurisdictions (European Food Safety Authority 2021a, 2021b; Xue et al. 2023). Environmental fate also warrants attention, including carrier degradation, waste streams, persistence, and potential release during manufacture and disposal. Consumer acceptance cannot be assumed: transparency about ingredients, demonstrated safety, affordability, cultural preferences, and clear nutritional benefits are essential for adoption. Therefore, nanoencapsulation should complement, not displace, established fortification, dietary diversification, and biofortification programs. Priority should be given to scalable, food‐grade carriers, independent long‐term safety assessment, human intervention trials measuring nutritional outcomes, and life‐cycle evaluation before widespread deployment (McClements 2020; Xue et al. 2023).

Nanotechnology applications in food science, nutrition, nutraceuticals, food microbiology, nutrient delivery, etc. have been linked to numerous benefits, including the nanotechnology applications in the nanoencapsulation of nutrients, phenolic compounds, antioxidants, antimicrobial agents, flavors, fish oil, essential fatty acids, and colorants for improved protection, stability, bioavailability, quality, controlled release, target delivery, and absorption. Nanoencapsulation and nanocarrier systems such as solid lipid nanoparticles, nanoemulsions, nanoliposomes, nature‐inspired nanocapsules and nanocarriers, biopolymeric nanocarriers, and nanostructured lipid carriers are applied for encapsulating vitamins, minerals, proteins, carbohydrates, phenolic compounds, antioxidants, antimicrobial agents, flavors, fish oil, essential fatty acids, and colorants (Pateiro et al. 2021; Awuchi, Morya, et al. 2022). The methods commonly used in the nanoencapsulation of these compounds include; coacervation, emulsification, and supercritical fluid (mainly for lipophilic and hydrophilic compounds); inclusion complexation, nanoprecipitation, and emulsification solvent evaporation (mainly for lipophilic compounds); electrospinning and electrospraying (mainly for hydrophilic compounds) (Zehra et al. 2021; Pateiro et al. 2021). More studies are required to improve and promote the nanoencapsulation of these compounds. Nanoencapsulation of nutrients, phenolic compounds, antioxidants, antimicrobial agents, flavors, fish oil, essential fatty acids, and food colorants have been shown to improve their protection, bioavailability, quality, supply, control release, target delivery, and absorption in the body (Awuchi, Morya, et al. 2022). Nanoencapsulation using carbohydrate‐based and protein‐based delivery system for food ingredients have been developed and present promising applications.

### 4.6 Biotechnology

Biotechnological applications can be well utilized to improve nutrient supply from foods and agricultural commodities. Biotechnology involves “the integration of natural sciences and engineering sciences in order to achieve the application of organisms, cells, parts thereof and molecular analogues for products and services”. Biotechnology has been widely applied in agriculture and can be developed to improve nutrient supply (Ranjha et al. 2022). Biotechnology firms can contribute to future food security by improving the nutrition and viability of urban agriculture and by improving the nutrient profile of the crops. Genetically modified crops (“GM crops”) are plants used in agriculture, the DNA of which has been modified with genetic engineering techniques (Wu et al. 2017). In most cases, the main aim is to introduce a new trait that does not occur naturally in the species. Although farmers in the developed and developing world have widely adopted GM technology, more still needs to be done, especially in the area of improving nutrient supply from foods and their bioavailability.

Biotechnology can improve the nutritional quality of staple foods through biofortification, genetic engineering, genome editing, and marker‐assisted breeding. These approaches may increase iron, zinc, provitamin A, or other nutritionally relevant compounds, potentially complementing conventional fortification and dietary diversification. However, increased nutrient concentrations do not necessarily translate into improved human nutritional status: bioavailability, food processing, habitual consumption, baseline deficiencies, and clinical outcomes must be evaluated. Evidence from human interventions is therefore more consequential than compositional improvements alone (Garg et al. 2018; Bouis and Saltzman 2017). At national and international levels, responsible implementation requires transparent risk assessment, regulatory oversight, ecological monitoring, and equitable access to seeds and intellectual property. Potential gene flow, biodiversity effects, and unintended agronomic consequences require context‐specific assessment. Ethical governance should include farmer participation, culturally informed public engagement, and safeguards against dependence on costly proprietary seed systems. Adoption also depends on locally adapted varieties, reliable markets, affordability, and extension support (European Food Safety Authority, Panel on Genetically Modified Organisms 2022). Accordingly, biotechnology should be judged not simply by technical performance, but by demonstrable nutritional benefit, environmental sustainability, and distributive justice.

Countries should embrace the application of biotechnology in food production. Biotechnology significantly helps to improve the quality of food and nutrients along with improving the quantity of food supply (National Academies of Sciences et al. 2016). Biotechnology can improve the sustainability of production systems by requiring fewer inputs to control pests and better protect the quality of water and land mass around us (Wu et al. 2017; Ranjha et al. 2022). Biotechnology can be used for the upgrading of traditional food processing based on fermentation such as the procedures used to produce gari, a fermented, gritty and starchy food derived from cassava. Biotechnology can also help to eliminate toxic components, including antinutrients, either by genetic engineering or through food processing.

### 4.7 Improving Agricultural Practices

Good agricultural practices should be encouraged and promoted globally. Healthy soils produce healthy crops that in turn nourish people and animals with the required nutrients. Indeed, soil quality is directly linked to food quality and quantity (López‐Yerena et al. 2019). Soils supply the essential nutrients, water, oxygen, and root support that our food‐producing plants need to grow and flourish. For individual nutrients, existing studies show that organic fertilization practices produce crops with higher levels of ascorbic acid, lower levels of nitrate, and improved protein quality compared with conventionally grown crops (López‐Yerena et al. 2019).

## 5 Matching Interventions to the Limiting Step

Interventions should be matched to the dominant bottleneck rather than selected because they are technologically novel. If the main limitation is low nutrient content, crop diversification, biofortification, or fortification may be appropriate (World Health Organization [WHO] 2022; FAO 2025). If the nutrient is present but bound within the matrix, fermentation, germination, milling choices, or meal composition may be more relevant. If intestinal absorption is impaired by disease or medication, food‐level interventions alone may be insufficient. Where the principal constraint is affordability or seasonal access, income support, school meals, market infrastructure, and social protection are more directly relevant. These categories overlap, but an explicit causal diagnosis helps prevent interventions from being judged against outcomes they cannot reasonably change.

### 5.1 A Limiting‐Step Framework for Nutritional Intervention

The effectiveness of a nutritional intervention depends not only on the quantity of a nutrient present in a food, but also on whether that nutrient can be released from the food matrix, remain chemically available during digestion, cross the intestinal epithelium, and subsequently be utilized by the body (Table 4). These processes are related but not interchangeable. Nutrient content represents the amount present in the food; bioaccessibility describes the fraction released and potentially available for intestinal absorption; bioavailability concerns the fraction absorbed and available for physiological utilization. Consequently, an intervention that increases nutrient concentration without addressing the process restricting its absorption may produce only a modest improvement in nutritional status. This distinction is particularly important for plant‐based foods, in which phytate, polyphenols, protein–mineral interactions, and structural characteristics can constrain nutrient release or absorption despite apparently adequate nutrient composition (Capuano and Janssen 2021; Ofori et al. 2022; Xia et al. 2026; Kaur et al. 2022; Elegbeleye et al. 2025; Ashraf 2025). A limiting‐step framework therefore requires nutritional interventions to be selected according to the dominant constraint in a particular food–nutrient–consumer combination. For example, phytate‐rich cereal foods may benefit from phytate degradation, whereas foods containing insufficient iron may require fortification or biofortification. If the food already contains adequate iron but its non‐haem fraction is poorly absorbed, combining it with an ascorbate‐rich food or selecting a more suitable fortificant may be more appropriate. Conversely, where the main constraint is poor intake, limited food access, inadequate dietary diversity, or low acceptability, a technically effective food‐processing intervention may have little practical nutritional impact. The evidence on biofortified crops, zinc‐fortified foods, and food processing illustrates why nutrient concentration, absorption, and improvements in human nutritional outcomes should be assessed separately rather than treated as equivalent indicators of success (Ofori et al. 2022; Xia et al. 2026; Kaur et al. 2022; Elegbeleye et al. 2025; Ashraf 2025; Nsabimana et al. 2024).

**TABLE 4:** Matching nutritional interventions to the dominant limiting step. The following evidence synthesis table links the principal constraint, its proposed mechanism, the intervention, and the outcomes needed to determine whether the intervention has worked. The evidence strength descriptions are qualitative interpretations of study design and outcome relevance.

Limiting step	Mechanism of limitation	Matched intervention	Target nutrient/food	Supporting evidence	Expected benefit	Critical limitation	Recommended evaluation (References)
Low initial nutrient content	Insufficient nutrient concentration limits the maximum amount deliverable per serving	Conventional fortification	Iron‐poor cereal flour	Human intervention and fortification evidence	Increased nutrient intake	Does not necessarily overcome poor absorption or low consumption	Nutrient content, intake, ferritin, hemoglobin (Kaur et al. 2022; Elegbeleye et al. 2025; Ashraf 2025)
Low crop nutrient density	Crop genotype or growing conditions constrain mineral accumulation	Agronomic or conventional biofortification	Iron‐ or zinc‐poor staple crops	Systematic review of biofortification	Increased intrinsic nutrient concentration	Increased crop content may not yield proportionate absorption	Crop assays, retention, fractional absorption, status (Ofori et al. 2022; Xia et al. 2026)
High phytate concentration	Phytate complexes reduce mineral solubility and absorption	Phytase treatment	Cereals and legumes	Human and food‐processing evidence	Greater mineral availability	Effects depend on pH, phytase activity, and residual phytate	Phytate, soluble mineral, human absorption (Nikolaou et al. 2026)
Phytate‐rich grain structure	Endogenous phytase activity may be insufficient under normal processing conditions	Controlled germination	Whole grains and seeds	Experimental processing research	Phytate degradation and potentially greater mineral accessibility	Germination conditions and nutrient losses vary	Phytate, mineral retention, bioaccessibility (Lemmens et al. 2018)
Phytate‐rich cereal or pulse matrix	Acidification and microbial enzymes can hydrolyse phytate	Lactic acid fermentation	Maize and other staple foods	Primary experimental study	Lower phytate and improved estimated mineral availability	In vitro or compositional changes do not establish human benefit	Phytate, mineral retention, absorption (Nsabimana et al. 2024)
Inadequate phytate reduction	Processing methods alone may not sufficiently activate phytase	Combined soaking, germination, and fermentation	Phytate‐rich maize	Primary experimental research	Greater phytate reduction than some single processes	Additional time, water, labor, and process control may be required	Nutrient retention, phytate, acceptability (Nsabimana et al. 2024)
Poor mineral release from grain	Mineral chelation persists after basic preparation	Optimized hydrothermal processing	Wheat and cereal products	Primary experimental study	Increased iron and zinc bioaccessibility	Conditions that improve release may affect other quality attributes	Mineral speciation and bioaccessibility (Lemmens et al. 2018)
Low non‐haem iron solubility	Inhibitory meal constituents restrict iron availability	Ascorbate‐rich food with the meal	Plant‐based iron sources	Human intervention reviews	Enhanced non‐haem iron absorption	Response varies with meal composition and baseline status	Iron absorption and longer term status (Loganathan et al. 2023)
Polyphenol‐related iron inhibition	Certain polyphenols bind iron and reduce its availability	Adjust meal composition or timing	Tea‐ or polyphenol‐rich diets with iron sources	Human and clinical‐trial synthesis	Potentially less inhibition during iron‐containing meals	Blanket restriction may reduce dietary diversity and beneficial polyphenol intake	Meal‐level absorption and dietary adequacy (Xu et al. 2021; Scarano et al. 2023)
Poorly available iron fortificant	Fortificant chemistry affects solubility and absorption	Select a suitable iron compound	Cereal‐based foods	Human iron‐absorption evidence	Greater absorbed iron per unit added	Bioavailability must be balanced against sensory effects, stability, and cost	Absorption, stability, sensory quality (Nikolaou et al. 2026)
Low zinc absorption	Dietary phytate complexes limit zinc availability	Zinc fortification	Commonly consumed staple foods	Systematic review and meta‐analysis	Improved zinc status in some populations	Co‐fortification complicates attribution of health effects	Plasma zinc, deficiency prevalence, intake (Kaur et al. 2022; Elegbeleye et al. 2025; Ashraf 2025)
Low zinc density in staple crops	Inadequate zinc content limits dietary supply	Zinc biofortification	Zinc‐poor staple crops	Systematic review of human studies	Increased zinc absorption from biofortified foods	Evidence on sustained status improvement remains limited	Total absorption, plasma zinc, dietary exposure (Ofori et al. 2022; Xia et al. 2026)
Poor protein digestibility	Food structure or molecular interactions limit proteolysis	Optimized thermal processing	Plant proteins and protein‐rich foods	Food‐protein digestibility reviews	Improved access to digestive enzymes	Excessive heating can cause aggregation or amino acid damage	Digestibility, amino acid availability (Loveday 2023)
Slow or incomplete protein digestion	Protein structure limits enzyme access and amino acid release	Controlled enzymatic hydrolysis	Protein ingredients	Mechanistic and in vivo processing evidence	Faster protein digestion and absorption	Faster absorption is not equivalent to greater total utilization	Amino acid kinetics and nitrogen utilization (Loveday 2023)
Matrix‐related nutrient entrapment	Physical structure controls release and digestion kinetics	Matrix redesign or suitable size reduction	Mixed foods and functional formulations	Mechanistic review	Improved nutrient release	Excessive structural disruption may alter satiety or food quality	Digestion kinetics and bioaccessibility (Capuano and Janssen 2021)
Processing‐related nutrient loss	Heat, milling, or storage reduces retained nutrient quantity	Optimize processing and storage	Biofortified staples and fortified foods	Systematic review of post‐harvest handling	Better retention of target nutrients	Retention is not proof of absorption or clinical benefit	Retention after processing and storage (Huey et al. 2023; Ceasar et al. 2026)
Inflammation‐related biomarker distortion	Acute‐phase responses alter interpretation of ferritin	Contextual interpretation with inflammatory markers	People at risk of iron deficiency	WHO guidance	More valid classification of iron status	Biomarkers remain imperfect and context dependent	Ferritin with inflammatory markers and hemoglobin (World Health Organization [WHO] 2020)
Insufficient dietary exposure	Low frequency or quantity of consumption limits total nutrient delivery	Improve coverage, availability, and sustained consumption	Fortified or biofortified staple foods	Effectiveness evidence from fortification studies	Greater population‐level nutrient exposure	Supply‐chain coverage does not guarantee individual intake	Coverage, compliance, consumption, status (Kaur et al. 2022; Elegbeleye et al. 2025; Ashraf 2025)
Poor acceptability or preparation feasibility	Sensory rejection or practical barriers reduce intervention uptake	Reformulate and co‐design with consumers	Fortified or processed foods	Food‐processing and effectiveness evidence	Improved sustained consumption	Acceptability is population‐specific and may change over time	Sensory testing, adherence, repeat consumption (Capuano and Janssen 2021)
Multiple concurrent constraints	Nutrient scarcity, inhibitors, poor access, and physiological factors interact	Combine food processing, nutrient addition, and delivery strategies	Staple‐based diets at risk of inadequacy	Integrated evidence across food and human studies	Potentially greater overall nutritional benefit	Synergy must be demonstrated; cost and unintended effects may increase	Nutrient retention, absorption, status, function, cost‐effectiveness (Ofori et al. 2022; Xia et al. 2026; Nsabimana et al. 2024)

*Note:* The table is a mechanistic synthesis, not a claim that every listed intervention has demonstrated clinical efficacy in every target population. “Primary experimental study” denotes experimental food processing evidence where specified; reviews and guidelines are identified separately. The citation attached to each row supports the intervention rationale but does not necessarily provide direct evidence for every proposed outcome.

### 5.2 Food Matrix Constraints: Improving Release Before Increasing Supply

Food matrix modification is most appropriate when a substantial proportion of a nutrient remains physically entrapped or chemically bound within the food. The matrix influences hydration, enzyme accessibility, molecular interactions, and the release kinetics of nutrients during gastrointestinal digestion. Processing can therefore improve or impair nutritional delivery depending on the food structure, the nutrient concerned, and the conditions applied (Table 4). Heating, for example, may improve the digestibility of plant proteins by inactivating certain antinutritional factors, but severe thermal treatment can also promote aggregation, cross‐linking, or reactions that reduce the availability of particular amino acids. The implication is that processing should not be classified as inherently beneficial or detrimental; it should be evaluated against a defined nutrient‐specific endpoint (Capuano and Janssen 2021; Loveday 2023). Phytate reduction provides a particularly informative example of matching an intervention to a limiting step. Phytate can form complexes with iron, zinc, and other minerals, although the magnitude of its effect depends on the food matrix and the presence of other interacting constituents. Soaking, germination, fermentation, and phytase treatment can reduce phytate under suitable conditions. In a 2024 study of maize, combining soaking, germination, and fermentation with Lactiplantibacillus plantarum reduced phytate by up to 85.6% and substantially lowered phytate‐to‐mineral molar ratios. However, these results establish improved compositional and estimated bioavailability indicators rather than demonstrating equivalent improvements in human mineral status. Such distinctions matter because laboratory measures of mineral solubility, in vitro digestion, and molar ratios cannot fully reproduce intestinal absorption, systemic regulation, or long‐term dietary exposure (Nsabimana et al. 2024; Zhang, Stockmann, et al. 2022). Fermentation should likewise be treated as a process whose efficacy depends on its operating conditions rather than as a universally effective nutritional solution. Microbial phytase activity, acidification, substrate characteristics, fermentation duration, and temperature may determine whether phytate degradation is sufficient to alter mineral availability. Recent human evidence concerning fermented bread suggests that improvements in acute iron absorption do not consistently translate into improved ferritin or total‐body iron over longer interventions. This discrepancy illustrates a central limitation of food‐based intervention research: a favorable mechanistic response is not necessarily a clinically meaningful outcome. Where phytate is the principal barrier, fermentation may be appropriate; where the initial mineral concentration is low, fermentation alone cannot compensate for the missing nutrient, and combining phytate reduction with fortification may be more rational (Nikolaou et al. 2026; Nsabimana et al. 2024).

### 5.3 Chemical Interactions: Matching the Intervention to Nutrient‐Specific Inhibitors and Enhancers

The chemical environment surrounding a nutrient can be as important as its total concentration. Non‐haem iron absorption, for instance, is influenced by ascorbate, phytate, and selected polyphenols, while zinc absorption is affected by phytate and the broader dietary matrix. This means that interventions should target the relevant interaction rather than assume that all antinutritional compounds exert the same effect or that one processing method will improve every mineral simultaneously. The phytate‐to‐mineral molar ratio can help characterize risk, but it is an incomplete predictor when other ligands, protein properties, and competing ions influence mineral solubility and absorption. Accordingly, nutritional assessment should combine compositional analysis with direct bioaccessibility or absorption measurements wherever feasible (Zhang, Stockmann, et al. 2022; Ofori et al. 2022; Xia et al. 2026). Ascorbate‐rich foods can improve non‐haem iron absorption by maintaining iron in a more soluble and absorbable form. However, the practical value of this strategy depends on the amount and timing of ascorbate intake, the composition of the meal, the individual's baseline iron status, and the durability of the dietary change. Evidence concerning vitamin C co‐administration with therapeutic iron also shows why food‐level enhancement and clinical treatment should not be conflated: a mechanistic rationale for improving absorption does not establish that adding vitamin C will always provide a meaningful additional hematological benefit when iron treatment is already effective. The appropriate intervention therefore depends on whether the objective is to enhance absorption from a habitual meal, prevent deficiency, or treat established iron‐deficiency anemia (Loganathan et al. 2023; Ofori et al. 2022; Xia et al. 2026).

Biofortification and conventional fortification address a different limiting step: insufficient nutrient concentration in the food supply. Biofortification increases nutrient content in crops through breeding or related agricultural approaches, whereas conventional fortification adds nutrients during food manufacture or preparation. Both can be effective, but neither guarantees adequate absorbed supply. For example, increasing zinc concentration in a staple crop may improve total zinc absorption and reduce the phytate‐to‐zinc ratio, yet the resulting improvement in zinc status depends on the quantity consumed, the residual inhibitory matrix, and the duration of exposure. Similarly, the effects of zinc‐fortified foods on health outcomes may be difficult to attribute to zinc alone when multiple micronutrients are co‐fortified (Awuchi, Atoki, and Echeta 2026; Awuchi, Iyiola, et al. 2026). Evaluation must therefore distinguish the intervention's direct nutrient effect from the effects of the food vehicle, co‐interventions, and changes in total dietary intake (Kaur et al. 2022; Elegbeleye et al. 2025; Ashraf 2025; Ofori et al. 2022; Xia et al. 2026).

### 5.4 Human‐Level Constraints: From Absorbed Nutrient to Nutritional Benefit

Even when food composition and intestinal absorption are optimized, human physiology can remain a limiting factor. Baseline nutrient stores, age, physiological requirements, inflammation, gastrointestinal function, and other aspects of health can alter the relationship between intake, absorption, circulating biomarkers, and tissue utilization. Inflammation, for example, can complicate the interpretation of ferritin because ferritin is an acute‐phase reactant; an apparently adequate ferritin concentration does not necessarily exclude ID in an inflammatory context. Likewise, a short‐term increase in serum iron after a meal cannot by itself establish that an intervention will improve hemoglobin, restore iron stores, or reduce the prevalence of deficiency. These considerations require researchers to select outcomes appropriate to the proposed mechanism and to interpret biomarkers in their physiological context (WHO 2020; Nikolaou et al. 2026). The final constraint is often behavioral and structural rather than biochemical. An intervention may improve absorption under controlled conditions but fail in practice if the food is expensive, culturally unacceptable, inconvenient to prepare, inconsistently available, or poorly consumed. Food fortification can have substantial population‐level value when implemented through widely consumed vehicles, but its effectiveness depends on coverage, compliance, quality control, and the suitability of the vehicle for the target population. Dietary advice to separate inhibitory beverages from iron‐rich meals, for example, may be mechanistically sound but less effective where meal patterns are constrained by work, school, household resources, or customary practices. These factors make intervention feasibility part of nutritional efficacy rather than a secondary implementation concern (Kaur et al. 2022; Elegbeleye et al. 2025; Ashraf 2025; Ofori et al. 2022; Xia et al. 2026).

### 5.5 An Integrated Decision Rule for Intervention Selection

The most justifiable strategy is to diagnose the dominant limiting step, select an intervention with a mechanism capable of addressing it, and measure outcomes at several levels. At minimum, studies should distinguish nutrient content, nutrient retention after processing, in vitro bioaccessibility, human absorption where feasible, relevant status biomarkers, and functional or clinical outcomes. A multi‐level assessment helps prevent the overinterpretation of surrogate outcomes and reveals whether a proposed strategy has failed because it did not change the food, did not improve absorption, or did not deliver a sufficient and sustained dose to the consumer. This distinction is particularly important for interventions involving biofortified crops, fermented foods, and novel processing technologies, where promising laboratory results may precede adequate evidence of long‐term effectiveness in humans (Ofori et al. 2022; Xia et al. 2026; Nsabimana et al. 2024; Nikolaou et al. 2026). Ultimately, matching interventions to limiting steps supports a shift from nutrient‐centric formulation towards outcome‐oriented nutritional design. The strongest intervention is not necessarily the one that maximizes nutrient concentration or produces the largest immediate change in a laboratory marker; it is the one that addresses the dominant constraint while preserving food quality, affordability, safety, acceptability, and sustained consumption. Where multiple constraints coexist, a combined strategy, such as phytate reduction plus fortification, or nutrient‐dense food provision plus measures to improve access, may be more effective than a single intervention. Nevertheless, combination approaches should be tested rather than presumed superior, because interactions among processing, nutrient forms, the food matrix, and human physiology may produce diminishing returns or unintended trade‐offs (Kaur et al. 2022; Elegbeleye et al. 2025; Ashraf 2025; Ofori et al. 2022; Xia et al. 2026; Capuano and Janssen 2021).

## 6 Emerging Challenges, Study Limitations, Comparative Appraisal of Major Interventions, Opportunities, Recommendations, Research Priorities and Reporting Standards

### 6.1 Emerging Challenges

#### 6.1.1 Translating Improved Nutrient Composition Into Meaningful Human Outcomes

A persistent challenge in nutritional intervention research is the assumption that increasing the concentration of a nutrient or improving its release during digestion will necessarily improve nutritional status. Nutrient delivery is a multistage process involving food composition, processing‐related retention, gastrointestinal bioaccessibility, intestinal absorption, physiological utilization, and sustained consumption. These stages are interdependent, but improvement at one stage does not establish success at the next. The food matrix can alter digestion and nutrient release, while host physiology determines how absorbed nutrients are distributed and utilized. Consequently, a formulation that demonstrates superior in vitro bioaccessibility should be considered promising rather than clinically effective until the effect is corroborated by appropriately designed human studies (Capuano and Janssen 2021). Recent evidence concerning cereal fermentation and iron nutrition illustrates this translational problem (Table 5). A 2026 systematic review of human intervention studies found that sourdough fermentation and phytate reduction could improve acute iron absorption under particular experimental conditions, yet the longer term evidence did not consistently demonstrate improvements in iron stores. Some favorable outcomes were observed when phytate reduction was combined with iron fortification, suggesting that removing an inhibitor may be insufficient when the absolute nutrient supply remains inadequate. This distinction challenges the use of phytate reduction or laboratory‐derived bioavailability indices as stand‐alone indicators of nutritional effectiveness. The appropriate endpoint depends on the intervention's purpose: food chemistry can establish mechanistic plausibility, absorption studies can test nutrient transfer, and longitudinal human studies are required to establish whether the intervention improves nutritional status (Nikolaou et al. 2026).

**TABLE 5:** Critical appraisal of emerging challenges, intervention opportunities, research priorities, and reporting requirements.

Challenge or limitation	Comparative appraisal	Opportunity	Recommendation	Research priority	Key outcome or indicator	References
Nutrient concentration is used as a proxy for nutritional benefit	Composition is necessary but does not establish absorption or utilization	Evaluate the complete nutrient‐delivery pathway	Separate compositional, absorption, and health outcomes	Link food‐level changes to human outcomes	Retention, absorption, status, function	Capuano and Janssen (2021)
Heavy reliance on in vitro bioaccessibility	Laboratory models simplify intestinal and systemic physiology	Use mechanistic screening to identify promising formulations	Confirm clinically relevant claims in human studies	Validate model predictions against human absorption	Agreement between model and human estimates	Nikolaou et al. (2026)
Inconsistent long‐term effects of fermentation	Acute absorption improvements may not persist as improved iron status	Optimize fermentation and combine it with nutrient addition where justified	Include adequate follow‐up and suitable controls	Test fermentation independently from fortification	Ferritin, hemoglobin, total‐body iron	Nikolaou et al. (2026)
Residual phytate and other inhibitors	Phytate is important but cannot explain all mineral interactions	Combine targeted processing methods where beneficial	Measure multiple inhibitors and nutrient retention	Establish food‐specific processing conditions	Phytate, mineral retention, absorption	Feizollahi et al. (2021)
Potential nutrient losses during processing	Antinutrient reduction may coincide with losses of minerals or bioactives	Optimize processing conditions for net nutritional value	Report nutrient mass balance before and after processing	Quantify trade‐offs across nutrients	Retention, degradation, sensory quality	Elliott et al. (2022)
Uncertain contribution of individual fortificants	Co‐fortification may obscure nutrient‐specific effects	Use factorial designs and informative comparators	Report the full fortification premix	Distinguish individual and combined effects	Biomarkers and prespecified functional outcomes	Tsang et al. (2021)
Variable effectiveness of zinc fortification	Biomarker improvement is better established than some functional outcomes	Improve targeting and monitoring of fortified food vehicles	Assess efficacy and program effectiveness separately	Evaluate sustained benefits in vulnerable groups	Plasma zinc, deficiency prevalence, health outcomes	Tsang et al. (2021)
Limited evidence on zinc‐biofortified foods	Increased absorption does not automatically demonstrate sustained status improvement	Develop biofortified crops suited to local diets	Test realistic consumption quantities and durations	Determine dose–response and long‐term effects	Total zinc absorption, zinc status	Gomes et al. (2023)
Polyphenol–iron interactions are oversimplified	Isolated compounds and whole‐food exposures are not equivalent	Refine meal‐level dietary recommendations	Avoid blanket restriction without evidence of net benefit	Examine dose, meal composition, and baseline status	Iron absorption and iron‐status markers	Xu et al. (2021)
Biomarkers are affected by physiological context	Inflammation can distort interpretation of ferritin	Improve diagnostic classification	Measure inflammation when indicated and interpret biomarkers contextually	Standardize status assessment across studies	Ferritin, hemoglobin, CRP, AGP as appropriate	WHO (2020)
Short intervention duration	Early biomarker responses may not reflect replenishment of nutrient stores	Conduct adequately powered longitudinal trials	Justify duration against the biological outcome	Determine the minimum duration for meaningful benefit	Change in status over time	Nikolaou et al. (2026), WHO (2020)
Heterogeneous study populations	Baseline status and dietary exposure can modify treatment effects	Identify groups most likely to benefit	Prespecify subgroup analyses and interaction hypotheses	Investigate age, deficiency, and dietary effect modification	Stratified treatment effects	Tsang et al. (2021)
Inadequate characterization of the food matrix	Differences in structure and composition impair cross‐study comparison	Develop standardized food characterization	Report matrix, raw materials, processing, and preparation	Improve reproducibility of digestion studies	Matrix descriptors and analytical methods	Capuano and Janssen (2021)
Poor comparability between interventions	Different mechanisms and comparators may make pooled effects misleading	Use direct comparative trials	Group evidence by mechanism and outcome	Compare processing, fortification, and combined approaches	Relative effect sizes and uncertainty	Nikolaou et al. (2026), Tsang et al. (2021)
Limited evidence on real‐world adherence	Controlled efficacy may not translate into sustained consumption	Integrate consumer research and implementation evaluation	Measure actual intake, adherence, and acceptability	Identify socioeconomic and cultural effect modifiers	Coverage, adherence, consumption	Tsang et al. (2021)
Affordability and implementation constraints	Biochemical effectiveness alone cannot establish population suitability	Optimize cost, supply chains, and food‐vehicle selection	Include economic and feasibility outcomes	Compare cost‐effectiveness across settings	Cost per beneficiary and nutritional outcome	Tsang et al. (2021)
Incomplete systematic‐review reporting	Missing search, selection, and appraisal details impede reproducibility	Standardize review reporting	Follow PRISMA 2020 and provide reproducible searches	Improve transparency and updateability	Checklist completeness, search reproducibility	Page et al. (2021)
Overstatement of evidence certainty	Statistical significance and reporting completeness do not establish confidence	Use structured certainty assessment	Evaluate risk of bias, inconsistency, indirectness, imprecision, and publication bias as applicable	Improve certainty grading and decision relevance	Outcome‐level certainty and justified conclusions	Cochrane (2026). Page et al. (2021)

*Note:* Key—AGP, α‐1‐acid glycoprotein; CRP, C‐reactive protein. *Interpretive note*—Rows 1–6 principally concern food composition and nutrient release; rows 7–12 focus on intervention effectiveness and human physiology; rows 13–18 address comparability, implementation, and evidence quality. This classification is intended to support decision‐making. The table translates the discussion into actionable recommendations for research design, evidence synthesis, and public health implementation. The citations at the end of each row support the principal evidence or methodological recommendation; the proposed priorities are analytical recommendations rather than claims that every strategy has already demonstrated clinical effectiveness.

#### 6.1.2 Interactions Between Bioactive Compounds and Essential Nutrients

Bioactive food constituents present a further challenge because their effects are context‐dependent rather than uniformly beneficial or detrimental. Polyphenols, for example, may bind non‐haem iron and reduce its absorption under certain dietary conditions, but their biological effects cannot be adequately characterized by their iron‐binding properties alone. A systematic review and meta‐analysis of randomized controlled trials examined the effects of dietary polyphenol supplementation on iron status and erythropoiesis, demonstrating the importance of evaluating measurable human outcomes rather than inferring nutritional harm solely from a proposed biochemical mechanism. This evidence cautions against indiscriminate recommendations to eliminate polyphenol‐rich foods, particularly when such foods contribute dietary diversity and other potentially beneficial compounds. The more justifiable approach is to distinguish the effects of isolated supplements from those of habitual foods and to evaluate dose, formulation, meal composition, duration, and baseline iron status (Xu et al. 2021; Ahmad et al. 2026). A related difficulty is that an intervention targeting one nutrient may produce different effects on other nutrients or food properties. Intensive processing may improve the accessibility of a target mineral while reducing the retention of heat‐sensitive compounds, changing texture, or increasing processing costs. Similarly, fortification can raise nutrient density without addressing inhibitory interactions in the food matrix. These trade‐offs require researchers to assess the overall nutritional quality of the resulting food rather than optimize a single nutrient in isolation. Food‐matrix research supports a more integrated model in which physical structure, nutrient interactions, digestion, and consumer exposure are evaluated together (Capuano and Janssen 2021).

#### 6.1.3 Heterogeneity in Human Responses and the Interpretation of Biomarkers

The same intervention may produce different outcomes across populations because nutrient requirements, baseline nutritional status, dietary patterns, inflammatory conditions, and gastrointestinal physiology differ. An improvement in nutrient absorption among individuals with depleted stores may not be reproduced among nutrient‐replete participants, while a short‐term change in circulating concentrations may not indicate a sustained improvement in body stores. Iron research particularly illustrates the importance of contextual biomarker interpretation: ferritin is informative about iron stores, but inflammation can elevate its concentration and complicate the classification of deficiency. The WHO therefore recommends interpreting ferritin in conjunction with relevant indicators of inflammation where appropriate. Studies that fail to account for these factors may misclassify participants, underestimate deficiency, or incorrectly attribute changes in biomarkers to the intervention (WHO 2020, 2022; FAO 2025). This heterogeneity also has implications for pooled effect estimates. Combining studies that investigate different nutrient compounds, doses, food vehicles, exposure durations, and participant groups can produce a summary estimate that is statistically calculable but clinically difficult to interpret. In some cases, apparent inconsistency reflects genuine effect modification rather than poor research quality; in others, it results from imprecise measurements, inadequate power, or incompatible outcome definitions. Accordingly, quantitative synthesis should be guided by biological and methodological comparability, not by the mere availability of numerical results. Where substantial clinical or methodological heterogeneity prevents a meaningful pooled estimate, a structured narrative synthesis may be more justified than an indiscriminate meta‐analysis (Page et al. 2021; Nikolaou et al. 2026).

### 6.2 Study Limitations and Implications for Evidence Interpretation

#### 6.2.1 Limitations in Study Design and Intervention Characterization

A major limitation across food‐based nutritional research is the difficulty of separating the effect of the target intervention from the effects of the surrounding food matrix, background diet, and concurrent nutritional exposures (Awuchi, Atoki, and Echeta 2026; Awuchi, Iyiola, et al. 2026). In fortification trials, for example, a product may contain several micronutrients, making it difficult to attribute observed improvements in nutritional status to one nutrient. In addition, differences in food vehicles, fortificant compounds, baseline dietary intake, and consumption frequency can undermine comparisons between otherwise similar studies. A systematic review and meta‐analysis of zinc‐fortified foods found improvements in plasma zinc concentrations and a lower prevalence of zinc deficiency, but several functional outcomes could not be attributed specifically to zinc because some interventions included multiple micronutrients. Such findings support fortification as a potentially useful public‐health strategy while highlighting the need for carefully designed comparisons that identify the contribution of individual nutrients and combinations (Tsang et al. 2021). A further limitation concerns the frequent reliance on surrogate endpoints. Reduced phytate concentrations, increased soluble mineral fractions, and improved in vitro digestion results can establish that an intervention changes a relevant mechanism, but these measurements cannot independently establish that the intervention prevents deficiency or improves health. Conversely, a human trial may fail to demonstrate a change in nutritional status despite a genuine improvement in absorption if the study is too short, the participants are not deficient, or the baseline diet supplies insufficient quantities of the target nutrient. These possibilities should be distinguished during evidence appraisal rather than interpreted collectively as intervention failure. The evidence on sprouting and cereal fermentation illustrates why researchers must report mechanistic and human outcomes separately and avoid treating bioaccessibility as a validated substitute for longer term nutritional benefit (Elliott et al. 2022; Nikolaou et al. 2026).

#### 6.2.2 Limitations in the Measurement of Bioavailability and Nutritional Status

Methods used to estimate bioavailability vary considerably in biological relevance and analytical precision (Table 5). Simulated gastrointestinal digestion, mineral solubility, and cellular uptake models can be valuable for screening food formulations, but they simplify processes involving intestinal transport, endogenous regulation, competing nutrients, and whole‐body nutrient requirements. Even stable‐isotope absorption studies, which provide more direct evidence of nutrient transfer, generally measure a specific exposure under defined conditions and may not predict the effects of habitual consumption over months. In vitro models are therefore best used as mechanistic tools that inform the selection of candidate interventions for human testing, not as replacements for human evidence when clinical effectiveness is the research question (Capuano and Janssen 2021; Nikolaou et al. 2026). Biomarker limitations create an additional source of uncertainty. The suitability of an outcome depends on the nutrient, the target population, the duration of exposure, and the physiological processes affecting the marker. For iron, hemoglobin alone cannot establish ID, while ferritin can be affected by inflammation. For zinc, the interpretation of circulating concentrations also requires attention to sampling conditions and the limitations of the biomarker. Consequently, a robust evaluation should use a prespecified set of outcomes that reflects both nutrient status and the intended clinical or functional benefit, with appropriate adjustment or stratification where necessary. WHO guidance on ferritin assessment provides an example of why biomarkers should be interpreted within a clearly defined diagnostic framework rather than as isolated laboratory values (WHO 2020; Tsang et al. 2021).

#### 6.2.3 Limitations in Evidence Synthesis and Generalizability

Systematic reviews inherit the limitations of their included studies. Inadequate randomization, incomplete reporting, small samples, selective outcome reporting, and short follow‐up may weaken confidence even when the pooled estimate is statistically significant. Moreover, differences in intervention design can make a single pooled effect misleading. For example, studies of iron fortification, phytate reduction, and fermentation may target different stages of nutrient delivery and use different comparators and outcome measures. Combining these studies without accounting for their distinct mechanisms risks obscuring which intervention works, for whom, and under what conditions. PRISMA 2020 improves transparency in reporting the review process, but compliance with reporting standards does not itself establish that the underlying evidence is unbiased or sufficiently certain (Page et al. 2021; Nikolaou et al. 2026). External validity is equally important. Evidence generated under controlled conditions may not translate directly to populations with different staple foods, cooking practices, food‐processing infrastructure, dietary diversity, or patterns of nutrient deficiency. Similarly, efficacy under supervised feeding does not necessarily predict effectiveness when participants purchase, prepare, and consume the intervention in everyday settings. The evidence for zinc fortification demonstrates the importance of evaluating both efficacy and effectiveness, while the limited human evidence on zinc‐biofortified crops underscores the need to establish how much of the improved nutrient supply translates into meaningful changes in status. Generalizability should therefore be assessed explicitly, rather than assumed from the statistical significance of an intervention effect in a particular population (Tsang et al. 2021; Gomes et al. 2023).

### 6.3 Comparative Appraisal of Major Interventions

#### 6.3.1 Processing, Fermentation, and Antinutrient Reduction

Processing interventions are most attractive when the limiting step is the release or accessibility of nutrients already present in a food. Sprouting, soaking, germination, fermentation, and enzymatic treatment can reduce phytate or alter food structure, but their effects depend on the food species, cultivar, processing conditions, and interactions with other constituents. Their principal advantage is that they may improve the nutritional value of familiar foods without requiring a separate fortification supply chain. Their limitations include variable efficacy, possible nutrient losses, requirements for water and energy, changes in sensory properties, and the need for adequate process control. Importantly, lower phytate content should not automatically be equated with improved nutritional status because the final outcome also depends on nutrient concentration, consumption, and physiological utilization (Elliott et al. 2022; Feizollahi et al. 2021).

#### 6.3.2 Fortification and Biofortification

Conventional fortification offers a means of increasing nutrient supply through foods processed at scale, whereas biofortification seeks to increase nutrient concentrations in crops through breeding or agronomic methods. Both strategies can reach large populations when their food vehicles are widely consumed and appropriately regulated. Evidence from zinc‐fortification studies indicates improvements in zinc biomarkers, although evidence for some functional outcomes remains less certain and co‐fortification complicates attribution. Evidence on zinc‐biofortified crops suggests that increasing crop zinc concentration can increase total zinc absorption, but uncertainty remains regarding the dose and duration required to improve population‐level zinc status. Thus, the choice between fortification and biofortification should be based on local dietary patterns, food‐processing systems, baseline deficiency, affordability, regulatory capacity, and evidence of sustained consumption rather than on nutrient concentration alone (Tsang et al. 2021; Gomes et al. 2023).

#### 6.3.3 Dietary Modification and Bioactive Component Management

Dietary modification attempts to improve nutrient supply by changing meal composition, food combinations, or consumption patterns. This approach may be relatively inexpensive and can preserve the wider nutritional value of a diverse diet, but its effectiveness depends on adherence, access to appropriate foods, and compatibility with established dietary practices. It is particularly important to avoid oversimplifying the role of bioactive compounds. Polyphenols can inhibit non‐haem iron absorption under certain conditions, but the effects of isolated polyphenol supplementation cannot be assumed to represent the effects of whole foods consumed within mixed diets (Xu et al. 2021; WHO 2020; Ahmad et al. 2026). Dietary recommendations should therefore be nutrient‐specific, proportionate to the evidence, and designed to minimize unnecessary restriction. Where dietary modification is insufficient to address a confirmed deficiency, it should not replace an indicated fortification or clinical intervention (Xu et al. 2021; WHO 2020).

#### 6.3.4 Why a Combined Approach May Be Preferable

A combined strategy is biologically plausible when more than one limiting step is operating. For instance, phytate reduction may improve the availability of iron or zinc, while fortification addresses insufficient absolute nutrient content. Yet the superiority of combined approaches should not be presumed. Processing may affect fortificant stability, mineral interactions, sensory acceptability, and production costs, while additional intervention components can make implementation and causal attribution more difficult. Comparative trials should therefore evaluate single‐component and combined strategies against appropriate controls and measure both nutritional benefit and implementation burden. The strongest evidence‐based recommendation is to select interventions according to the dominant constraint, then test whether additional components provide an incremental benefit that justifies their costs and potential trade‐offs (Nikolaou et al. 2026; Tsang et al. 2021; Feizollahi et al. 2021).

### 6.4 Opportunities, Recommendations, and Research Priorities

#### 6.4.1 Opportunities for More Effective Nutritional Strategies

The most important opportunity is to integrate food science, human nutrition, agricultural research, clinical evaluation, and implementation science within a common intervention framework. Rather than developing foods solely to maximize nutrient concentration, researchers can characterize the limiting mechanism first and design the intervention around it. This approach can support more targeted use of phytase, fermentation, fortification, crop biofortification, and dietary modification, while reducing the likelihood of investing in interventions that improve laboratory measurements without addressing the actual cause of low nutrient supply. The food‐matrix literature and evidence from zinc fortification support this shift towards evaluating both mechanistic plausibility and demonstrated nutritional outcomes (Capuano and Janssen 2021; Tsang et al. 2021). An additional opportunity lies in the development of standardized, sequential testing pipelines. Candidate interventions could first undergo compositional and processing assessments, followed by in vitro digestion studies, appropriate human absorption experiments, and adequately powered longitudinal trials. This sequence would allow researchers to identify ineffective approaches earlier while ensuring that promising formulations are evaluated against meaningful health outcomes. Such a pipeline should not become a rigid requirement for every study: established fortification approaches may already warrant population‐level evaluation, whereas novel processing methods may require more preliminary characterization. The essential principle is that the level of evidence should match the strength of the proposed claim (Nikolaou et al. 2026; Page et al. 2021).

#### 6.4.2 Recommendations for Researchers and Public Health Practitioners

Researchers should define the primary nutritional outcome before selecting the intervention, identify the proposed limiting step, and specify the evidence needed to demonstrate that the constraint has been overcome. Studies should report the composition of the food before and after processing, nutrient retention, relevant inhibitors and enhancers, fortificant form, preparation procedures, actual consumption, and background dietary exposure. Human studies should additionally report baseline nutritional status, adherence, relevant confounders, follow‐up duration, adverse events, and prespecified outcomes. These details are essential for distinguishing intervention failure from inadequate exposure, inappropriate endpoint selection, or insufficient follow‐up (Page et al. 2021; Tsang et al. 2021). Public health practitioners should prioritize interventions that combine demonstrated nutritional benefit with acceptable cost, food safety, feasibility, and population reach. Where staple foods are processed centrally and consumed consistently, fortification may be appropriate; where nutrient‐poor crops dominate diets, biofortification may provide a complementary approach; and where antinutritional factors are a major constraint, targeted processing may improve nutrient utilization. These options should be adapted to the local context and monitored for unintended effects, including excessive exposure in groups not at risk of deficiency. Nutritional policy should therefore combine evidence of efficacy with surveillance of dietary exposure, nutritional status, implementation quality, and equity (WHO 2020; Tsang et al. 2021).

#### 6.4.3 Priority Research Questions

Future research should address three interconnected questions: whether the intervention changes the intended mechanism, whether that change improves nutrient absorption and status, and whether the intervention remains effective and feasible under real‐world conditions. Studies should investigate effect modification by baseline deficiency, age, physiological requirements, dietary pattern, and relevant inflammatory conditions. They should also determine whether improved nutrient delivery is sustained over time and whether benefits extend to functional outcomes rather than remaining confined to biochemical markers. The limited and sometimes inconsistent longerterm evidence for fermentation‐based iron interventions provides a clear rationale for this type of research (Nikolaou et al. 2026; WHO 2020). A further priority is the development of comparative studies that evaluate interventions against one another, rather than comparing each intervention only with a different control. Direct comparisons of optimized processing, fortification, biofortification, dietary modification, and combined strategies would help establish their relative effectiveness, cost, and feasibility. Where direct trials are not practical, carefully justified indirect comparisons may be useful, provided that important differences between populations, interventions, and outcomes are acknowledged. Evidence synthesis should distinguish the quantity of evidence from its certainty and relevance, so that research gaps are not concealed by large but heterogeneous study counts (Tsang et al. 2021; Page et al. 2021).

#### 6.4.4 Reporting Standards and Methodological Transparency

Systematic reviews should follow PRISMA 2020 to report eligibility criteria, information sources, search strategies, screening procedures, data extraction, risk‐of‐bias assessment, synthesis methods, and study flow. The PRISMA checklist is a reporting framework rather than a substitute for rigorous methods; a review can be fully reported yet still be limited by a poorly specified question, incomplete searches, inappropriate study selection, or weak evidence appraisal. For reviews that include both food‐processing experiments and human intervention studies, the eligibility criteria should explicitly distinguish these evidence categories and specify how each contributes to the review's conclusions (Page et al. 2021). Primary studies should provide enough detail to support replication and critical appraisal. For randomized trials, this includes allocation procedures, comparator characteristics, intervention dose and duration, adherence, outcome definitions, missing data, and statistical methods. Food‐processing experiments should additionally describe the food matrix, raw‐material characteristics, processing conditions, analytical methods, and the basis for any bioaccessibility calculations. Reviews should assess risk of bias and, where appropriate, use a structured approach such as GRADE to evaluate certainty across outcomes. The conclusions should remain proportionate to the evidence: improved compositional properties are not equivalent to improved absorption, and improved absorption is not automatically equivalent to improved health (Page et al. 2021; Cochrane 2026; Nikolaou et al. 2026).

## 7 Conclusion

Low nutrient supply emerges from interacting processes rather than one universal cause. The review indicates that intervention choice should follow diagnosis: food‐matrix constraints may warrant preparation or processing changes; population‐level deficiencies may justify monitored fortification; malabsorption or disease requires clinical assessment; and food‐access constraints require social protection and food‐system action. Fortification can improve intake efficiently where suitable vehicles reach the target population, but it cannot replace dietary diversity or treatment of disease and must be monitored for coverage, nutrient stability, excessive intake, and inequitable reach. Similarly, postharvest interventions should be evaluated using commodity‐ and region‐specific loss data, not generalized percentages. Nanotechnology and biotechnology remain promising in selected applications, but scale‐up should depend on demonstrated human benefit, safety, regulatory clarity, affordability, environmental consequences, and public acceptance. Future studies should connect food‐composition and in vitro outcomes to human absorption, biomarkers, clinical endpoints, program coverage, and cost‐effectiveness. A practical priority is to combine local diagnosis with interventions that are evidence‐based, affordable, culturally acceptable, and monitored for both benefits and unintended effects.

## Author Contributions

**Chinaza Godswill Awuchi:** conceptualization, investigation, writing – original draft, methodology, validation, visualization, writing – review and editing, software, formal analysis, project administration, data curation, supervision, resources.

## Funding

The author has nothing to report.

## Conflicts of Interest

The author declares no conflicts of interest.

## Data Availability

Data sharing not applicable to this article as no datasets were generated or analyzed during the current study.
